# Cathelicidins: Opportunities and Challenges in Skin Therapeutics and Clinical Translation

**DOI:** 10.3390/antibiotics14010001

**Published:** 2024-12-24

**Authors:** Lenka Dzurová, Edita Holásková, Hana Pospíšilová, Gabriela Schneider Rauber, Jitka Frébortová

**Affiliations:** Czech Advanced Technology and Research Institute (CATRIN), Palacký University Olomouc, 77900 Olomouc, Czech Republic; edita.holaskova@upol.cz (E.H.); h.pospisilova@upol.cz (H.P.); jitka.frebortova@upol.cz (J.F.)

**Keywords:** cathelicidins, clinical trials, wound healing, skin drug delivery, recombinant production, peptide formulations, resistance mechanism

## Abstract

Cathelicidins are a group of cationic, amphipathic peptides that play a vital role in the innate immune response of many vertebrates, including humans. Produced by immune and epithelial cells, they serve as natural defenses against a wide range of pathogens, including bacteria, viruses, and fungi. In humans, the cathelicidin LL-37 is essential for wound healing, maintaining skin barrier integrity, and combating infections. Cathelicidins of different origins have shown potential in treating various skin conditions, including melanoma, acne, and diabetic foot ulcers. Despite their promising therapeutic potential, cathelicidins face significant challenges in clinical application. Many peptide-based therapies have failed in clinical trials due to unclear efficacy and safety concerns. Additionally, the emergence of bacterial resistance, which contradicts initial claims of non-resistance, further complicates their development. To successfully translate cathelicidins into effective clinical treatments, therefore, several obstacles must be addressed, including a better understanding of their mechanisms of action, sustainable large-scale production, optimized formulations for drug delivery and stability, and strategies to overcome microbial resistance. This review examines the current knowledge of cathelicidins and their therapeutic applications and discusses the challenges that hinder their clinical use and must be overcome to fully exploit their potential in medicine.

## 1. Introduction

Antimicrobial peptides (AMPs), also known as host defense peptides (HDPs), are innate immune response components present across all organisms. The Antimicrobial Peptide Database (APD) represents a comprehensive source of information about antimicrobial peptides (AMPs), providing data on classification, structure, function, and activity. It categorizes peptides into natural, predicted, and synthetic groups, with information collected manually over the past 20 years from reliable sources. The last update (December 2023) of the APD showed a total of 3940 entries, of which 3146 were natural AMPs from the six life kingdoms (383 bacteriocins/peptide antibiotics from bacteria, 5 from archaea, 8 from protists, 29 from fungi, 250 from plants, and 2463 from animals), while 190 were predicted and 314 were synthetic AMPs. The entries retrieved from the database show antibacterial, antibiofilm, anti-endotoxin, antiviral, antifungal, antiparasitic, anticancer, anti-diabetic, wound healing, anti-inflammatory, insecticidal, synergistic, and other activities [1]. Among the natural AMPs with known antimicrobial activity, 373 are present in mammals, of which 144 are human host defense peptides (hHDPs). The hHDPs encompass well-known families such as cathelicidin (LL-37), defensins, histatins, and dermcidin [2].

Cathelicidins represent a group of cationic peptides with amphipathic properties produced by various immune system cells, as well as by epithelial cells in the skin and mucosal surfaces. They play a crucial role in the innate immune response of many vertebrates, including humans and animals, serving as natural defense mechanisms against various pathogens such as bacteria, viruses, and fungi [3]. Their diverse biological properties make cathelicidins promising candidates for therapeutic applications via topical administration. For example, numerous studies have shown that AMPs are upregulated throughout all stages of wound healing. Many of them also work on maintaining the integrity of the skin barrier and its function, which gives them the potential to treat other skin conditions such as melanoma, acne, diabetic foot ulcers, and psoriasis [4]. Several peptides have failed phase III clinical trials in practice due to unclear efficacy or lack of superiority over conventional treatments. These failures may arise from efforts to convert natural AMPs into drug candidates without fully understanding how they target and kill bacterial cells. To facilitate effective clinical application, several factors must be addressed, including high development costs, cytotoxicity, reduced activity in clinically relevant environments, and the emergence of bacterial resistance, despite initial claims that resistance would not develop [5].

In this review, we look at the information available in the vast scientific literature related to cathelicidins and their analogues. We aim to identify the challenges that need to be overcome to boost their clinical application in skin therapeutics. From our perspective, the main roadblocks increasing the gap between basic research and the translation of cathelicidins to therapeutics are their susceptibility to protease degradation, the high production costs, the microbial resistance development, and the limited understanding of disease- and tissue-specificity before the design of proper formulations for drug delivery. We have structured this review with a brief introduction about cathelicidins and their applications, followed by a discussion of clinical trials and a critical assessment of issues related to manufacturing, biopharmaceutics, and pharmacological aspects of antimicrobial resistance. To finalize, the above-mentioned aspects are briefly brought together to identify new directions and perspectives that could benefit the successful implementation of cathelicidins in dermatological clinical practice.

## 2. Cathelicidins

Cathelicidins are produced as precursor proteins capable of releasing AMPs after proteolytic cleavage [6]. These peptides are secreted upon leukocyte activation as inactive precursors (pre-pro-peptides) and stored inside secretory granules of neutrophils and macrophages. The highly conserved N-terminal pro-domain, which includes the so-called cathelin domain, is proteolytically cleaved to release a highly variable C-terminal domain in the form of mature bioactive peptides [7]. Due to their considerable variability in amino acid sequences and sizes (usually 12–80 amino acid residues with a maximum of 100 residues), mature cathelicidins display significant diversity in their three-dimensional structures [8,9]. The C-terminal domains of certain precursor cathelicidin proteins exhibit α-helical structures, while others feature β-hairpin conformations, and some possess proline/arginine-rich motifs. According to this, cathelicidin peptides can be categorized into five groups: (a) cyclic dodecapeptides, with a single disulfide bond (e.g., bovine bactenecin, sheep P54230); (b) porcine protegrins, featuring two disulfide bonds; (c) linear peptides, adopting an α-helical structure (e.g., bovine myeloid antimicrobial peptides BMAP-27, -28, and -34, human LL-37, etc.); (d) peptides rich in tryptophan (e.g., indolicidin) or containing high levels of proline-arginine residues (e.g., proline-arginine-39); and (e) short molecules organized in tandem repeats, such as bactenecins (e.g., bovine bactenecins Bac5 and 7, etc.) [3].

The structural conformation of cathelicidins possesses crucial elements necessary for their diverse functionality [10]. For instance, the rabbit CAP18_106–137_ domain exhibits a rigid α-helical conformation when interacting with lipid A, part of the lipopolysaccharide (LPS) of the outer leaflet of gram-negative bacteria, through either coulombic interaction with the diphosphoryl groups or hydrophobic interaction with the fatty acyl chains [11]. Human cathelicidin LL-37 adopts an amphipathic α-helical conformation in the region between residues 2 and 31 and a random C-terminal structure (residues 32–37) when interacting with membranes and their mimetics [12,13]. This structure was first described in sodium dodecyl sulfate micelles as a curved amphipathic helix–bend–helix pattern followed by a disordered C-terminal tail [13]. The hydrophobic part of the amphipathic helix is separated by a hydrophilic serine residue (S9) into two domains: an N-terminal short helix and a long central helix [14]. Such a structure explains the cooperative binding of LL-37 to LPS in the outer bacterial membrane. It also indicates that the central hydrophobic helix represents the main region of LL-37 responsible for antimicrobial activity [14]. In addition, S9 is important for peptide activity since its mutation reduces the antibacterial activity of LL-37 against *Escherichia coli* [12,15]. The hydrophobic region (represented by 35% of residues) is responsible for LL-37 oligomerization in aqueous salt solutions and for peptide–membrane interactions. Interestingly, oligomerization in physiological conditions reduces the ability to permeabilize bacterial membranes due to the preferred interaction of LL-37 with other molecular surfaces offering favorable hydrophobic characteristics. Therefore, the antimicrobial and immunomodulatory activity is highly medium- or environment-dependent [16].

A basic search (AMP Name Query: Cathelicidin) in the APD [1] identified 163 natural and 4 synthetic AMPs classified as cathelicidins (Table 1). A more detailed search uncovered 25 additional synthetic derivatives or hybrids of cathelicidins. Some cathelicidins were discovered in bovine granulocytes, including bactenecin (a dodecapeptide), proline-rich peptides (Bac5 and Bac7), and α-helical bovine myeloid antimicrobial peptides BMAP-27, BMAP-28, and BMAP-34 [17]. Additionally, numerous cathelicidins were identified in various mammals such as goats, pigs, sheep, and others. Interestingly, while many mammals express multiple cathelicidin genes, humans [7], mice, and some others express only one. Of the 90 mammal cathelicidins in the APD, 9 correspond to the human antimicrobial peptide LL-37 and its fragments generated from the precursor human cationic antibacterial protein of 18 kDa (hCAP18).

The proteolytical activity of endopeptidases such as serine proteases usually mediates the release of mature AMPs from precursor proteins. Moreover, it has been confirmed that proteases continue to cleave the AMPs, either generating shorter fragments that can possess antimicrobial activity or fully degrading the peptides [20,119]. For instance, LL-37 is primarily released from hCAP18 by the serine protease proteinase 3 in neutrophils [120]. Alternatively, kallikreins 5 (KLK5) and 7 (KLK7) in epidermal keratinocytes can also cleave LL-37 from hCAP18, but unlike proteinase 3, they continue to cut LL-37 into shorter peptides with antimicrobial activity, potentially leading to full peptide degradation [20]. It was later confirmed that LL-37 can also be released by other KLKs, such as KLK8 or KLK14 [119]. KLK5, KLK7, KLK8, and KLK14 belong to the kallikrein-related peptidases (KLKs, kallikrein-like kallikreins), which display trypsin- or chymotrypsin-like activities and are mostly localized in particular cells of the human skin, where KLK5, KLK7, and KLK14 are the most abundant [121].

### 2.1. Antimicrobial Activity of Cathelicidins

Cathelicidins display broad-spectrum activity against various microorganisms, including bacteria, fungi, protozoa, and viruses. The antimicrobial action is especially important in human skin diseases such as atopic dermatitis, psoriasis, rosacea, or acne. In the case of atopic dermatitis lesions, the level of cathelicidins is significantly reduced. This is associated with relapses of different infections as these peptides can suppress *Staphylococcus aureus*, herpes simplex virus, and vaccinia virus, which often colonize the skin of individuals with atopic dermatitis [122,123].

Target specificity of cathelicidins arises from differences in the cell membrane composition of pathogens compared to host cells [124]. Cationic AMPs, such as cathelicidins, preferentially bind to the negatively charged membranes of microbial pathogens, which are enriched with anionic components like LPS in gram-negative bacteria, lipoteichoic acid in gram-positive bacteria, and phosphomannan in fungi [124,125,126]. Their antibacterial effects can occur through membrane-disruptive actions, such as permeation or perforation leading to intracellular leakage, or through membrane penetration to exert intracellular effects. These mechanisms enable rapid microbial killing without targeting specific molecules or pathways, thereby minimizing the likelihood of pathogens developing resistance to cathelicidins and other AMPs [124].

Three principal models of membrane-pore formation are well described in the literature regarding the membrane-mediated antibacterial activity of AMPs [127,128]. In the barrel–stave model, AMPs aggregate and insert into the cell membrane bilayer as multimers, aligning parallel to the phospholipids to form a channel [127]. In the toroidal pore model, in turn, the AMPs embed vertically in the cell membrane and bend to create a ring-shaped pore. For instance, protegrins, representatives of cathelicidins, use both modes of action. Moreover, molecular dynamic simulations confirm that hairpin protegrin-1 can form stable octameric β-barrels and tetrameric arcs (half-barrels) in both implicit and explicit membranes [129,130,131]. The aggregate model represents a particular form of the toroidal pore model, where AMPs attach to the negatively charged cytoplasmic membrane, compelling the peptides and lipids to assemble into a peptide–lipid complex micelle [132,133]. The carpet model is mediated through AMP accumulation on the membrane surface and disruption of the cell membrane in a detergent-like fashion [124,127]. Representative of this mechanism of action is human cathelicidin LL-37 [134], or AMPs with a β-sheet structure [135,136].

The alternative mechanism of antibacterial action involves penetration of AMPs into the cytoplasm and interaction with intracellular components. This includes inhibiting the synthesis of nucleic acids (indolicidin [137]) and proteins (proline–arginine-rich AMPs PR-39, Bac5 [138]), enzyme activity (LL-37 [139]), and cell wall synthesis [124]. The translocation mechanisms, however, remain unclear and may vary depending on the peptide and bacterial species [133].

In addition to their antibacterial properties, cathelicidins also demonstrate antiviral potential against various viruses. Destabilization of the viral envelope through direct contact, virion damage, or inhibition of infection represents a common mechanism of action against enveloped viruses (e.g., LL-37 against influenza virus or Kaposi’s sarcoma-associated herpesvirus) [140,141]. On the other hand, they also exhibit activity against non-enveloped viruses such as rhinoviruses via reducing viral replication [142]. The antifungal activity of cathelicidins (e.g., LL-37, chicken cathelicidin CATH-2) is mostly mediated via cell membrane permeabilization and simultaneous vacuolar expansion leading to a decrease in cell size. The amount of internalized cathelicidins, below the minimal fungicidal concentration, can even affect the integrity of the nuclear envelope [143].

### 2.2. Immunomodulatory Activity of Cathelicidins

In addition to the antimicrobial activity, cathelicidins possess immunomodulatory properties that play crucial roles in shaping the host immune response. These peptides can promote barrier repair, produce chemokine and cytokine, and modulate the activity of immune cells, such as leukocytes, neutrophils, and antigen-presenting cells (macrophages, dendritic cells), thereby influencing innate and adaptive immune responses. The immunomodulatory effects of cathelicidins are influenced by environmental stimuli, cell and tissue type, interactions with cellular receptors, and peptide concentration [144].

The primary immunomodulatory mechanism of cathelicidins involves the direct recruitment of immune cells through cathelicidins’ chemoattractant properties (LL-37, porcine PR-39) or indirectly via the induction of chemokine release [144], mediated by various cellular receptors such as chemokine receptors CCR6 or CCR2, G protein-coupled receptors, and formyl peptide receptors (by, e.g., cationic HDPs, represented by human LL-37 and chicken CATH-2) [133,145,146], as well as interactions with intracellular proteins like glyceraldehyde-3-phosphate dehydrogenase and sequestosome 1 (ubiquitin-binding protein p62) (e.g., human LL-37, chicken CATH-2) [133,147]. These effects are context-dependent, varying with the phase of infection and inflammation, as demonstrated by LL-37’s role in mediating CXCR2 internalization on monocytes and neutrophils, which subsequently reduces chemotaxis [148]. Neutrophils, as key immune effector cells during early-phase infections, are the primary source of cathelicidins, releasing them in granule form during degranulation. To control the infection, peptides such as LL-37 can enhance the influx of neutrophils by direct chemotactic function or inducing the secretion of the neutrophil chemokines Interleukin (IL)-8 and GRO-α in a mitogen-activated protein kinase-dependent manner [149,150]. Interestingly, the exogenous application of cathelicidins (e.g., LL-37, CATH-2, BMAP-28) or other synthetic peptides leads to the regulation of inflammation in various animal models of infection and sepsis [144].

Moreover, the AMPs link innate and adaptive immunity through the ability to interact with the antigen-presenting cells mainly with monocytes, macrophages, and dendritic cells, and induce their relocalization to the infection site. They boost phagocytosis in macrophages and lymphocyte response [151]. For instance, LL-37 is altering adaptive immunity by affecting the function and differentiation of dendritic cells in vivo [152] and in vitro [151] or eventually enhancing the activation and proliferation of B cells [153]. On the other hand, the murine analogue of LL-37, known as cathelicidin-related antimicrobial peptide (CRAMP), can directly affect T- and B-cell responses, and thus promote and regulate humoral and cellular antigen-specific adaptive immune responses [138].

Interestingly, AMPs are involved in balancing inflammation to maintain immune homeostasis. For instance, the anti-inflammatory function of peptides was confirmed by Putsep et al. They observed that patients with morbus Kostmann, a condition marked by severe neutropenia, frequently suffer from periodontal disease as a consequence of hCAP18/LL-37 deficiency and lower levels of α defensins in neutrophils [154]. Remarkably the absence of a murine CRAMP in CRAMP-deficient mutant mice leads to heightened inflammatory responses in comparison to wild-type mice [155]. Depending on the microenvironment and concentration, cathelicidins can modulate inflammation by serving as either a pro-inflammatory or anti-inflammatory agent. LL-37 is typically expressed in neutrophils, mast cells, macrophages, and monocyte granules under normal conditions. Nevertheless, its expression may increase in keratinocytes and other epithelial cells due to infection, injury, or inflammation [156]. Cathelicidins’ anti-inflammatory effects involve preventing the absent in melanoma 2 (AIM2) inflammasome formation and reducing interferon-γ (human LL-37), tumor necrosis factor -α (human and bovine cathelicidins), IL-4, and IL-12 levels [144,157,158]. On the other hand, the LL-37’s pro-inflammatory actions involve decreasing IL-10 levels; increasing IL-1β, IL-12p40, and IL-18 levels; inducing type I interferons in plasmacytoid dendritic cells and keratinocytes; and promoting mast cell degranulation followed by the release of inflammatory mediators [157,159,160,161,162].

Cathelicidins also play a role in psoriasis, a disease characterized by abnormal proliferation and differentiation of keratinocytes and T cells, and excessive activation of plasmacytoid dendritic cells, leading to high levels of pro-inflammatory cytokine and chemokine production [122]. Numerous AMPs, such as human β-defensins (hBDs), LL-37, S100A proteins, and RNase 7, are overexpressed in psoriatic keratinocytes, potentially acting as both antimicrobial and anti-inflammatory agents. For instance, hBDs enhance the keratinocyte expression of anti-inflammatory cytokine IL-37 [163], or LL-37 blocks the release of inflammatory cytokine IL-1β via inhibition of inflammasome activation [162].

In contrast to psoriasis, where AMP helps to defeat the infection and inflammatory response, the pathophysiology of rosacea involves chronic inflammatory and vascular responses, with altered innate immune responses. These include abnormal production of LL-37, increased activity of KLK5, and elevated levels of LL-37 fragments in lesional skin leading to inflammation [164,165]. Even LL-37 fragments stimulate innate immunity by promoting leukocyte recruitment, angiogenesis, and pro-inflammatory cytokine production. Mechanisms leading to LL-37 overproduction include vitamin D and toll-like receptor 2 pathways. Targeting KLK5 activations may help prevent abnormal LL-37 processing and reduce inflammation in rosacea [122,164].

### 2.3. Wound Healing Properties and Administration of Cathelicidins to the Skin

The combination of anti-inflammatory and antimicrobial activities makes cathelicidins great candidates for wound healing. The process of restoration of injured tissues is characterized by four distinct overlapping phases: hemostasis, inflammation, proliferation, and remodeling [166]. Immediately upon injury, many inflammatory cells migrate to the wound site to combat the infection. This is followed by the proliferative phase, marked by the formation of granulation tissue, epithelialization, and angiogenesis. In the final remodeling phase, the new collagen matrix becomes cross-linked and organized. AMPs may affect this repair process by inhibiting collagen production in dermal fibroblasts, as observed in the case of LL-37 [167]. Additionally, it has been shown that LL-37 recombinantly produced in *E. coli* stimulates endothelial cell proliferation, migration, and tubule-like structure formation, as well as enhanced vascularization and re-epithelialization in mouse trauma experiments [168].

Excessive levels of AMPs, however, can cause chronic inflammation, as demonstrated in Section 2.2 for skin diseases like psoriasis, rosacea, and atopic dermatitis [154]. The critical balance of activity and safety, therefore, may be mitigated by the selection of appropriate dosage forms, the identification of protease-rich target sites in the skin, and the understanding of tissue- and disease-specific factors that may affect the delivery and the overall performance of cathelicidins.

Successful topical treatment with cathelicidins depends, among other factors, on the specific characteristics of the skin. The skin is structured in three layers of different compositions that are named after their depth in the tissue, i.e., epidermis, dermis, and hypodermis [169]. These structures vary significantly among different species, genders, and body locations, as illustrated in Table 2. Porcine skin is the closest model to human skin, especially in concerning the thickness of the layers and the composition of the *stratum corneum*—although the skin appendixes vary slightly between both models [170,171]. Additionally, skin thickness, hydration, and aspect (i.e., furrows) may change with age [171,172]. For example, infants and elders tend to present thinner skin on some body sites in comparison to young adults [173,174,175]. Histological variations may also arise between male and female individuals, depending on the body location (i.e., males tend to show thicker skin) [171,172]. Importantly, in terms of wounds, the high content of androgen hormones like testosterone and their activity in the male skin has been reported to be detrimental to the regeneration of the tissue [176].

The stratification of the skin results in a contrasting environment between the lipophilic and low-diffusivity nature of the outermost layers of the skin, and the hydrophilic, matrix-abundant, and vascularized environment of the dermis [181,182]. This characteristic challenges the delivery of biopharmaceuticals to the skin [183]. Lipophilic molecules, for instance, tend to accumulate in the *stratum corneum* and find difficulties in partitioning towards the dermis. Amphiphilic peptides like cathelicidins, in turn, show impaired permeation through the *stratum corneum* and facilitated diffusion through the dermis [184,185]. This behavior is detrimental to the application of peptides in inflammatory and infectious skin diseases with no infiltrates. In the case of wound management, however, the physiopathology of the condition (i.e., the disruption of the barrier effect) favors the transport of foreign peptides and precursor proteins toward the sites of action. These are protease-rich layers of the skin, which may impair the kinetics of the AMP activity [186].

In healthy skin, kallikreins are mostly restricted to the epidermis and the hair follicles. Although their gene expression is spatial-specific (e.g., KLK6 is found strictly in the granular layer while KLK1 is also found in the basal layer of the epidermis), these proteins are able to broadly distribute throughout the tissue as a consequence of the cell migration that occurs during keratinization. A similar situation is also seen in the event of tissue damage or infection. In this case, kallikreins are transferred from the epidermis to the wound bed either via passive diffusion or with the aid of local blood circulation [187,188]. Cells from the hair follicle are also recruited in re-epithelialization processes and may be a source of kallikreins to the affected skin [189]. This means that precursor proteins can be easily cleaved into AMPs in wounds. The elevated exudate levels and distribution of proteases in the damaged tissue result in a quick cleavage of precursor proteins into AMPs and further consumption of the peptides—a fact that endorses the need for technological strategies to improve the pharmacokinetics and the stability of peptide formulations at the site of action [178,184].

## 3. Clinical Applications and Trials

### 3.1. Clinical Trials of Cathelicidin-Based Therapies

To date, clinical trials have only been conducted for human (synthetic or recombinant LL-37), bovine (synthetic indolicidin), and pig (synthetic protegrin-1) cathelicidins and their derivatives (Table 3) [190]. In addition, several dozen synthetic and hybrid forms of perspective cathelicidins have been prepared and tested for possible future biotechnological purposes or clinical applications.

Various topical formulations containing cathelicidins have been developed and tested in clinical trials, showing promising results. For instance, the application of LL-37 has effectively eradicated methicillin-resistant *S. aureus* (MRSA) and other antibiotic-resistant bacteria in preclinical and clinical trials. Among the peptides derived from LL-37, AMP60.4Ac (also known as OP-145; length 24 amino acids) was clinically tested in a randomized double-blind placebo-controlled multi-center phase II study for the treatment of chronic suppurative otitis media in adults. Evaluation of efficacy and safety in this study strongly supports the ongoing clinical development of AMP60.4Ac [192]. Moreover, creams and gel with AMP60.4Ac were tested against MRSA using in vitro epidermal and bronchial epithelial models, where cathelicidin formulated in hypromellose gel was stable and highly efficient against MRSA in both tested conditions [208]. LL-37, under the name Ropocamptide, was tested in clinical trials by Promore Pharma, Solna, Sweden (until 2016 named Lipopeptide AB) for the treatment of hard-to-heal leg ulcers (Table 3). The clinical trial demonstrated that LL-37 is safe and well tolerated when applied locally to nonhealing lower leg ulcers alongside standard compression therapy. Furthermore, a low dose of LL-37 may enhance healing in large, hard-to-heal venous leg ulcers [194,195]. Another synthetic LL-37 derivative, SAAP-148 (length 24 amino acids), showed enhanced antimicrobial activities against multidrug-resistant bacteria compared to the natural peptide due to improved cationicity and helicity of the peptide [209]. An example of a hybrid synthetic peptide is RP557 (17 amino acids long), derived from human cathelicidin LL-37, tachyplesin 1 (natural peptide from horseshoe crab), and the synthetic D2A21 (derived from *Drosophila cecropin*) [210]. RP557 has a broad-spectrum of antibacterial, antifungal, and antibiofilm effects with low cytotoxicity [210] and can increase susceptibility to other antibiotics when coadministered [211]. Additionally, capsules with recombinant LL-37 have been tested for COVID-19 treatment [193], and LL-37 injections have been administered to induce an antitumor response in melanoma patients (Table 3).

The most intensively studied cathelicidins for further pharmacology applications are synthetic peptides derived from bovine indolicidin. As such, omiganan pentahydrochloride (MBI-226) was tested during a variety of safety-based and efficacy studies and as a potential cure for facial seborrheic dermatitis, atopic dermatitis, genital warts, papulopustular rosacea, severe acne vulgaris vulvar, as an intraepithelial neoplasia cure, and for the prevention of central venous catheter-related bloodstream infections (Table 3). The omiganan treatment was mostly developed by BioWest Therapeutics Inc., Vancouver, BC, Canada (defunct) and Cutanea Life Sciences company, Wayne, PA, USA, (licensed omiganan from BioWest and acquired by Biofrontera, Leverkusen, Germany, in 2019).

Dusquetide, another indolicidin-derived synthetic peptide of five residues, has been tested as the active ingredient in the intravenous formulations SGX945, for the treatment of Behçet’s disease; SGX942, for the healing of severe oral mucositis; and SGX943, for combating bacterial infections by company Soligenix, Inc., Princeton, NJ, USA. Interestingly, the peptide does not possess direct antimicrobial activity but rather acts as an innate defense regulator (IDR), modulating the innate immune response to infection and tissue damage towards anti-inflammatory, anti-infective, and tissue healing responses [212]. Since IDRs do not possess direct antimicrobial activity, the probability of resistance development is reduced. They can be administered to patients with suspected bacterial infections before detailed diagnosis, used in combination with other antibiotics, as well as a treatment for antibiotic-resistant bacteria. Dusquetide also accelerates tissue repair following damage from trauma, chemotherapy, or radiation therapy. Its preclinical efficacy and safety have been demonstrated in numerous animal models against mucositis, colitis, macrophage activation syndrome, and bacterial infections [213]. Soligenix is currently conducting clinical research with SGX942 in oral mucositis in head and neck cancer patients (Table 3). Since SGX942 and SGX943 share the same active ingredient and formulation, many of the findings in the SGX942 trial program can be used to advance the SGX943 program. A phase II study evaluating the potential utility of SGX945 in treating aphthous ulcer flares in Behçet’s disease started in 2024 (Table 3).

Unlike Dusquetide, the clinical trials of other indolicidin-based treatments for catheter-associated urinary tract infections caused by *S. aureus* [214], topical skin anti-sepsis, and rosacea [197] were not successful. However, the trials for acne, atopic dermatitis, and vulvar intraepithelial neoplasia, are still in progress [201] (Table 3).

Intrabiotics Pharmaceuticals, Inc., Mountain View, CA, USA (since 2006 Ardea Biosciences, San Diego, CA, USA; 2012 bought by AstraZeneca, Cambridge, United Kingdom; 2018 closed) was developing three formulations of an iseganan-based drug (a synthetic analogue of bovine protegrin-1): a rinse for the potential treatment of mucositis, an aerosolized liquid for the potential treatment of respiratory infection, and a gel formulation for the potential treatment of pneumonia [215]. In patients receiving stomatotoxic chemotherapy, treatment with oral iseganan failed to prevent or reduce stomatitis or ulcerative oral mucositis [207]. It also showed no benefit in reducing the severity of ulcerative oral mucositis during radiotherapy for head-and-neck malignancy [216]. In addition, aerosolized iseganan caused a higher rate of pneumonia and mortality. As a result, Intrabiotics abandoned the development of iseganan-based drugs for this particular indication [217] (Table 3).

Spexis company, Allschwil, Switzerland (established in 2021, after merging Polyphor with EnBiotix) is testing inhaled Murepavadin, a novel bovine protegrin-1-derived drug, for the treatment of *Pseudomonas aeruginosa* infections in people with cystic fibrosis (Table 3); a phase III clinical trial of Murepavadin on patients with nosocomial pneumonia was however terminated due to a higher occurrence of acute kidney injuries [218].

### 3.2. Clinical Trials Focused on the Modification of Endogenous LL-37 Levels

Aside from the direct administration of cathelicidins, clinical studies have also focused on the endogenous production of AMPs. For example, vitamin D3 stimulated the expression of several AMPs, including hCAP18 [219]. Increased production of LL-37 in human skin by the biologically active form of vitamin D3 (1,25-dihydroxyvitamin D3) was demonstrated in vitro [219] and in vivo [220]. Oral vitamin D supplementation increases lesional and nonlesional levels of LL-37 in the skin of patients with atopic dermatitis [221,222]. According to in silico analysis, the hCAP18 promoter is activated by the binding of vitamin D to the corresponding receptor [223]. Microorganisms such as *Mycobacterium tuberculosis* stimulate the expression of vitamin D receptor and vitamin D-1-hydroxylase genes, resulting in increased production of the biologically active form of vitamin D3, which upregulates LL-37 expression [224].

Considering common vitamin D insufficiency, further clinical studies are needed to determine whether vitamin D supplementation offers a therapeutic approach to enhance the host’s ability to combat infections [225]. Unfortunately, a study of the effect of high-doses of vitamin D3 in smokers and non-smokers with and without HIV was terminated (trial ID: NCT03270709), and a survey of the role of vitamin D supplementation in active tuberculosis, focusing on LL-37 production, was withdrawn (trial ID: NCT00788320). The results of an older clinical trial, NCT00023335, which focused on the relationship between vitamin D, tuberculosis, and LL-37 without vitamin D supplementation, showed no relation between the level of vitamin D and concentrations of LL-37 in serum [226], which could have been the cause of failure in the above-mentioned tests as well. On the other hand, a study on the effect of vitamin D on patients with gum diseases has been completed for phase II (trial ID: CTRI/2017/08/009193).

Several clinical trials focused on periodontal health have been conducted, such as the study of salivary LL-37 and periodontal health in children exposed to passive smoking (trial ID: NCT03639376), including the effect of chlorhexidine and essential oil mouthwashes on hBD-2 and LL-37 saliva levels (trial ID: NCT04946617), and an observational study of LL-37 levels in the gingival crevicular fluid and the saliva of smokers and non-smokers with stage III and IV periodontitis (trial ID: NCT04861493).

Other clinical trials have investigated the inhibitory effects of topical ivermectin on serine protease activity, LL-37 production, and the skin microbiome of individuals with rosacea and during rosacea-specific inflammation (trial ID: NCT02806414), or evaluated the effect of topical aminocaproic acid in inhibiting KLK5 serine protease activity and LL-37 production during rosacea-specific inflammation (trial ID: NCT01398280). Most clinical trials involving LL-37-based drugs are in phase II, either ongoing or completed, as summarized in Table 3.

## 4. Challenges for Implementing Cathelicidin-Based Therapies in Clinics

Apart from the reasons for clinical trial failures mentioned previously, challenges such as high production costs, delivery system formulation, susceptibility to proteases, the development of microbial resistance, and pro-inflammatory roles limit cathelicidins’ clinical development. These challenges represent the most important points that need to be considered for the further clinical usage of these AMPs and are explored in more detail in the following sections.

### 4.1. Challenges Associated with the Production and Formulation of Cathelicidins

#### 4.1.1. Alternatives for the Production of Cathelicidins

One of the critical points for AMP clinical usage is their production in amounts suitable for further application. Although AMPs can be isolated from their natural sources, the tissue-specific production, the necessity for proteolytic activation, and the small quantities found in tissues are unsuitable for large-scale production [227]. The two alternative approaches include chemical synthesis and heterologous expression. Chemical synthesis offers the advantage of a high-purity product; various chemical modifications, such as C-terminal amidation or N-terminal acylation; and the possibility of incorporating D-amino acids or other unnatural residues, to increase in vivo stability against proteolytic degradation [228]. However, it presents an environmental risk due to using toxic chemicals. In addition, the synthesis of some peptides, such as those containing more than 35 residues or certain amino acids, may be difficult [229].

Compared to chemical synthesis, recombinant DNA technology represents a more sustainable, scalable, and cost-effective way to produce AMPs [230]. Both prokaryotic and eukaryotic systems can be used to express AMPs, each having specific advantages. Bacteria offer fast, high-level, and low-cost expression, while yeast and fungi also efficiently secrete the product into the culture media, making purification easier. Additionally, yeast, fungi, and insect cells provide some level of post-translational modification and are free from lipopolysaccharide endotoxins. The disadvantages of eukaryotic cells include higher expression costs, especially with insect cells, and longer expression periods. Molecular farming, using plants to produce proteins of pharmaceutical interest, has emerged as a new platform for the cost-efficient production of AMPs [231].

#### 4.1.2. Strategies for Microbial Production

Bacteria and yeasts represent the most common expression systems used for AMP production [227]. Due to the immense availability of genetic tools, rapid growth, and simple cultivation techniques, the majority of AMPs are expressed in the gram-negative bacterium *E. coli* as a host, although there are several examples of expression in gram-positive bacteria such as *Bacillus subtilis*, having the advantage of not containing any endotoxins and allowing secretion of the produced AMPs into media [229,232]. Yeasts combine the simplicity of a unicellular organism with the ability to perform most of the post-translational modifications required for a biologically active AMP. *Saccharomyces cerevisiae* and *Komagataella phaffii* (formerly *Pichia pastoris*) are the most commonly used yeasts to express recombinant AMPs [232]. A large number of vectors, strains, protocols, and physiological and genetic information are available for *S. cerevisiae*, making it a suitable host. However, the peptide yield is low due to ethanol production by fermentation. In comparison, *K. phaffii*, an obligate aerobic yeast that can use methanol as a carbon source, allows higher production of heterologous peptides due to the use of a strong methanol-inducible AOX1 promoter controlling the expression of the alcohol oxidase gene [233].

Since AMPs are inherently toxic to bacterial hosts due to their interaction with membranes, they are produced as inactive protein fusions and then processed in vitro to release the active AMP. In addition, fusion carrier proteins often improve expression levels by increasing cytoplasmic solubility and peptide stability towards proteolysis. The choice of a fusion tag may affect expression levels even if the same expression host cells are used. For example, the expression level of small ubiquitin-like modifier (SUMO)-LL-37 fusion was significantly lower than that of thioredoxin-LL-37 fusion [234,235]. Commercial expression plasmids offer various fusion tags, including thioredoxin, SUMO, glutathione S-transferase, maltose-binding protein (MBP), intein, or calmodulin-binding peptide, each of which has been successfully used to express various AMPs, including cathelicidins [227,229]. In many cases, two or more different tags are used: typically, one facilitating simple affinity chromatography purification, such as the histidine tag consisting of six or more histidine residues, and the other enhancing the solubility and/or stability (e.g., SUMO, MBP, or thioredoxin). Many fusion tags that improve solubility and stability can also facilitate the purification of peptide fusions using affinity chromatography methods. However, the use of affinity matrices contributes to the high costs of peptide production, and new tags allowing chromatography-free purification are being developed. These include the fusion of peptides to elastin-like polypeptides (ELP) [236] or the four-helix bundle biosurfactant protein DAMP4 [237]. ELPs are characterized by the presence of artificial repetitions of the pentapeptide motif with a VPGXG sequence where the X is any amino acid except proline [236]. ELPs can reversibly switch between soluble and aggregate forms depending on temperature allowing simple separation of fusion proteins from contaminants by increasing the temperature to the physiological range to precipitate ELP fusion protein. The subsequent decrease in the temperature solubilizes the fusion protein. The helix bundle structure of the DAMP4 protein remains stable and soluble under very high temperatures and sodium sulfate concentrations that disrupt cells and precipitate cellular proteins [238]. The properties of ELP and DAMP4 protein are retained when used as carriers for the production of AMPs, allowing their cost-effective purification [237,239,240]. An innovative use of ELP tags for peptide delivery was reported recently by Colomina-Alfaro et al. [241]. Indolicidin was fused to a human ELP, which contains both hydrophobic elastin-like sequences and the elastin-derived cross-linking domains, enabling cross-linking of the biopolymer chains to produce a stable hydrogel matrix. The susceptibility of human ELP to elastase activity can be exploited to create smart stimuli-responsive materials with antimicrobial properties that can be activated on demand [241].

The antimicrobial activity of peptides is, however, often negatively affected by the presence of a fusion tag [239,242]. To solve this problem, specific amino acid sequences cleavable by chemicals or enzymes are engineered into the expression plasmid to connect fusion partners. The most common chemicals to release the peptide are cyanogen bromide cleaving after methionine residue and acids, such as formic, trifluoroacetic, chloric, and acetic, cleaving between aspartate and proline residues [243]. The cleavage at recognition sites consisting of one or two residues can be rather unspecific, and the conditions of low pH and increased temperatures can compromise the peptide stability and introduce chemical modification of amino acids. Another alternative is represented by the inducible self-cleavage activity of intein tags that can be removed by thiol reagents or pH shift [244]. A photocleavable protein tag was recently used to express several AMPs [245]. Compared to chemicals, enzymatic methods offer more specific cleavage under mild conditions. The most commonly used proteases to release peptide from the fusion protein are enterokinase, factor Xa, thrombin, and tobacco etch virus protease, each recognizing different short sequence motifs [230]. Efficient cleavage requires a high ratio of proteases to proteins, long incubation times, and accessibility of the cleavage site to protease. As the cleavage by acid and some proteases leaves one or two extra amino acid residues at the peptide, assessment of antimicrobial activity after the recovery of the peptide from the fusion protein is necessary [230]. In addition, some proteases cleave occasionally at other sites, depending on the conformation of the protein substrate. A special case is a very specific cleavage of the SUMO tag, which is cleaved by an SUMO protease recognizing the tertiary structure of SUMO and cleaving at the end of the C-terminus of SUMO [230,235].

In the process of using tags, an additional purification step is required to separate the AMP from the cleaved affinity tag and the protease used. This can be addressed by utilizing tags used for the initial purification of a fusion protein or the strategic addition of other tags, most often His-tag, to either fusion protein or proteases. Immobilized metal-affinity chromatography is then used to bind His-tagged fragments and proteases after the cleavage step, while the AMPs are collected from the flow-through fraction [246,247]. Alternatively, other purification techniques that take advantage of specific properties of individual AMPs, such as their size, hydrophobicity, ionic properties, isoelectric point, and solubility, can also be employed. The minimum solubility of the proteins and peptides at their isoelectric point was used for simple and low-cost recovery of AMPs from other components of the cleavage mixture [237,243].

The production yield of AMPs can vary significantly due to factors such as the choice of host, expression plasmid, and fusion tag, the efficiency of the purification process and peptide cleavage from the fusion partner, and the properties of the peptide itself. As a result, no single strategy can be applied to achieve high expression and efficient purification of any peptide, and they have to be evaluated individually. Nevertheless, small-size fusion tags are more advantageous because the AMP represents a substantial part of the fusion protein, enhancing peptide yield. In addition to the already discussed properties of fusion tags, the expression levels can be considerably improved by codon optimization, the use of host strains containing tRNAs for rare codons, tandem multimeric expression, and hybridization expression strategies [248]. In the strategy of tandem multimeric expression, gene copies of the AMPs in the host cells are multiplied to increase the gene transcription level. At the same time, multimerization can reduce the toxicity of AMPs to expressing cells [249]. However, this does not always prove successful, as shown with the expression of indolicidin concatamers in *E. coli*, which was only possible using fusion to thioredoxin [249]. Similarly, the extracellular expression of tandem repeat multimers containing different number of copies of the LL-37 gene in *K. phaffii* inhibited cell growth, as the peptide was lethal to host cells. The lethal effect was avoided by targeting the expression to peroxisome, but the extraction was inefficient [250]. On the other hand, reptile cathelicidin-BF was successfully overexpressed in *K. phaffii* using cathelicidin-BF sequences connected in series through a short acid-cleavable linker [251]. In the hybridization expression strategy, two AMPs, usually with different properties, are fused to generate a new AMP with high expression yield, increased antimicrobial activity, and low cytotoxicity against host cells. The hybrid peptide combining the N-terminal amphipathic α-helix fragment of cecropin A with the core antimicrobial fragment of LL-37 possessed significantly greater antibacterial activity against a wide range of pathogens and displayed nearly no cytotoxicity compared to the parent peptides [252]. When produced in *E. coli* using the pET-SUMO vector, around 17 mg/l of pure active hybrid AMP was obtained [247]. Additionally, a hybrid of LL-37 fragment (2–31) and hBD-129 was successfully expressed in *E. coli* using the pET vector; it demonstrated significant antimicrobial effects [253]. A dimeric antimicrobial peptide consisting of human LL-37 and bovine indolicidin (both belonging to the cathelicidin family) separated by a flexible linker exhibited strong antimicrobial activity, salt ion stability, and low hemolytic activity when expressed in *K. phaffii* [254]. A similar strategy allowing the expression of recombinant AMPs that are non-toxic to host cells and resistant to proteolytic cleavage without using non-functional carrier protein is based on multidomain polypeptides combining several functional domains, not only with antimicrobial activity in a single protein [255]. The approach can be combined with targeted expression in inclusion bodies, stable protein nanoparticles produced during protein expression consisting mainly of the accumulated protein of interest. Although inclusion bodies are generally considered to be deposits of inactive proteins, they can often be used as a source of active soluble protein using mild extraction under non-denaturing conditions without any renaturation procedure [255]. In addition, inclusion bodies containing AMPs can slowly release the peptides, allowing for their direct therapeutic application, to maintain constant antimicrobial levels for extended periods [255].

#### 4.1.3. Strategies for Plant-Based Production

The expression of AMPs in plants has been shown to serve two different purposes, either plant protection from plant pathogens (not reflected in this review) or production for therapeutic use through molecular farming [256]. Among different expression systems, plants exhibit several advantages over bacterial and yeast expression systems, such as proper folding, glycosylation, and disulfide bond formation, critical for the antimicrobial activity of some peptides. In addition, plants are considered safe platforms for producing therapeutically relevant proteins and peptides, since plants are free of human and veterinary pathogens. Although the costs associated with development are high compared to microbial expression platforms, once established plant-based systems offer cost-efficient production, and easy, fast, and high-capacity scale-up, as well as low-cost purification and storage [256].

Various plant-based expression platforms are available, ranging from cell or tissue cultures to transgenic plants, offering either transient or stable expression after the transformation of nuclear or chloroplast genomes [256]. Different types of in vitro cultures of plant cells or tissues, such as cultures of suspension cells, calli, and hairy roots are useful for expressing recombinant proteins. The advantages of these systems include simplicity of transformation, product homogeneity, and the short time required to accumulate heterologous product (generally days or weeks after transformation) [256]. Suspension plant cell cultures represent promising platforms to produce various important biologically active products as they are grown in controlled environments under monitoring and defined conditions for growth. The production costs for recombinant proteins using plant suspension cell cultures are relatively low compared to bacterial systems as they require simple growth media and nutritional requirements [257]. The major disadvantages of suspension cultures are their relative instability, low protein productivity, and limited scale-up capacity. Moreover, cell cultures are very susceptible to contamination and overgrowth by microbes.

Stable transgenic lines generated by transgene integration into plant nuclear or chloroplast genomes produce recombinant peptides in subsequent generations, allowing easy scale-up and low-cost production. Transient expression is achieved via extrachromosomal gene expression within plant cells and provides high protein levels in a few days. After product recovery, freshly transformed plants are used for the next round of production [256,258]. Only a few cathelicidins have been expressed in plants, most of them by stable expression. Sheep cathelicidin SMAP-29 was stably expressed in *Nicotiana tabacum*, purified from leaf extracts using the intein-mediated self-cleavage mechanism, and shown to be active against *E. coli* [259]. The gene coding for LL-37 or its variant was integrated into the genome of Chinese cabbage [260], tomato [261], rice [262], and barley [242], but only the expression in barley was implemented with the goal of recovering the peptide. In other cases, LL-37 expression was reported to enhance resistance against various plant pathogenic organisms in respective transgenic plants. Protegrin-1, a cathelicidin forming disulfide bonds, was expressed in *N. tabacum*, both stably and transiently [263,264]. Stable expression in chloroplast resulted in the accumulation of protegrin-1, up to 26% of total soluble protein, and protected the plant from *Erwinia carotovora* infection [263]. The extract from transiently transformed tobacco plants decreased the cell viability of different mammalian pathogens [264]. It is important to note that the use of low-alkaloid *N. tabacum* eliminated the need to purify the recombinant protein from the extract to prevent alkaloid-related effects during antimicrobial assays.

Similar to microbial expression systems, AMPs expressed in plants exhibit intrinsic instability due to their small size, short half-life, low expression, and a certain level of cytotoxicity to plant hosts. The strategies to overcome the limitations include the fusion of the nucleotide sequence of the peptide to sequences of a proper promoter, subcellular targeting signals, and a carrier protein fulfilling diverse functions. Although heterologous expression of peptides in the whole plant body driven by constitutive promoters such as the cauliflower mosaic virus CaMV 35S promoter in dicots and maize ubiquitin-1 promoter in monocots enables high-level accumulation of recombinant product, the peptide expression in all tissues may negatively affect the growth and development of the transgenic plants. Targeting the peptide to specific tissues and organelles using tissue-specific promoters and proper sorting sequences can mitigate the phytotoxic effect of AMPs in host plants, improve peptide stability, and facilitate its isolation and purification. In this aspect, plant seeds, such as cereal grains or legume seeds, provide an inert and stable environment for peptide accumulation and deposition [265], and can be grown, harvested, and stored at low cost. In practical terms, the possibility of long-term seed storage at ambient temperature allows product processing on demand. A combination of the approaches described above was used to produce LL-37 in barley: the expression under the control of the endosperm-specific promoter of barley B1 hordein gene with concurrent incorporation of the N-terminal secretion sequence and C-terminal endoplasmic reticulum retention sequence KDEL targeted the peptide to protein bodies in endosperm of barley grains. The fusion of LL-37 with maltose-binding protein protected the peptide from long-term degradation and facilitated affinity purification [242,266]. Virtually any strategy using carrier proteins or hybrid and multidomain AMPs already described for microbial expression can also be applied in plants. One of the plant-specific approaches is represented by targeting the peptide to the surface of lipid storage organelles, called oil bodies, present in seeds. This can be accomplished by fusing the peptide with the oleosin protein under the control of the oleosin gene promoter [267]. Targeting peptides to oil bodies results in the accumulation of expressed peptides and simplifies product recovery, as oil bodies can be easily separated from other cellular components by flotation centrifugation. Another strategy uses fusion tags such as Zera, the proline-rich N-terminal domain of γ-zein derived from maize, hydrophobins, and ELPs, which can induce the formation of protein bodies containing a high concentration of the desired protein in transgenic plants [268]. These protein bodies are surrounded by a membrane protecting the recombinant protein from proteolytic degradation. Again, the fusion tags also facilitate the recovery of fusion proteins, using simple chromatography-free methods. As such they are suitable for sustainable production of AMPs by molecular farming.

#### 4.1.4. The Development of Target-Specific and Extended-Release Formulations

As a rule of thumb, the physicochemical descriptors needed to achieve passive transport of molecules from the *stratum corneum* to deeper layers of the skin are low molecular weight (<500 Da), octanol/water partition coefficient LogP ranging from 1 to 3 (i.e., moderate lipophilicity), and a melting temperature below 200 °C (i.e., indicating a low number of inter- and intramolecular interactions) [169,269]. From this list, it is evident that, in general, peptides show unfavorable characteristics for skin permeation. The strategies that can be employed to disrupt and/or overcome the skin barrier include the use of chemical enhancers (e.g., surfactants, fatty acids, terpenes, and sulfoxides) [270], vesicular or particulate carriers [184,269,271], novel delivery systems (e.g., microneedles) [272], and external physical stimuli (e.g., iontophoresis) [273]. Fumakia and Ho, for example, have demonstrated that solid lipid nanoparticles made from glyceryl monostearate with phosphatidylcholine could encapsulate LL-37-serpin A1 and accumulate into the skin [274]. Nanofibers of poly(ethylene-oxide) prepared via electrospinning, in turn, were used to control and retain LL-37 on wounds via the preparation of dressings or mats to be applied on the skin [275]. Such nanomaterials can be used for either extending the release or improving the diffusion of cathelicidins via the intercellular and intracellular routes of skin delivery—but not only this.

It has been reported, for instance, that skin appendages (e.g., hair follicles and sebaceous glands) play a significant role in the delivery of nanoparticles and macromolecules to vascularized regions of the dermis [276]. This route acts like a natural duct transporting particles through the *stratum corneum* and storing them for extended activity. The targeted applications are both topical (e.g., acne vulgaris and rosacea) and systemic activities (e.g., vaccination) [277,278,279]. Interestingly, the hair follicles present resident kallikreins that can activate cathelicidins into their antimicrobial fragments [187,188]. Although it has not yet been explored, this strategy could be useful in treating infectious and inflammatory diseases affecting the skin. The use of the follicular route to support the management of wounds is, however, not recommended because optimal formulations targeting the follicle tend to dehydrate the skin [280].

In the case of wound healing, one of the most important attributes of a formulation is the promotion of hydration [186]. This is generally attained by the use of polymeric matrices that form supramolecular networks and films [281]. Hydrogels, for instance, are ideal vehicles for tissue healing because of their high water content, biocompatibility, and porous arrangement, which facilitates the incorporation of actives and the production of commercial wound dressings [282,283,284]. In fact, according to Raileanu et al., the majority of the late-clinical-trial investigations of FDA-approved antimicrobial peptides administered topically were formulated as gels [285]. However, the choice of gelling agent crucially affects the product properties.

Different types of polymers have been used in the preparation of hydrogels and other formulations containing cationic peptides, including cathelicidins. These polymers include chitosan [286], polyacrylic acid (e.g., Carbopol 974 NF^®^) [287], hydroxyethyl cellulose [288], poly-2-hydroxyethyl methacrylate [289], methacrylamide [290], and poly(L-lactic acid) with its poloxamer block copolymer (e.g., Pluronic L35^®^ and F127^®^) [291]. Pluronic F127^®^ and Poly(ε-caprolactone) electrospun nanofibers were used to encapsulate the cathelicidin 17BIPHE2, a derivative of LL-37. This nanofiber dressing has sustained the release of the peptide for at least 28 days [292]. Poloxamers are especially interesting in wound healing because they provide thermo-responsive platforms able to form in situ gels with good product adhesion and coverage of the the ill tissue [293]. However, polymers are utilized not only for drug delivery but also for minimizing water loss and defending against external aggressors. They may also be used in the purification and the isolation of peptides—a crucial step in the preparation of biopharmaceuticals. A cross-linked sodium alginate/gelatin hydrogel, for example, was used to harvest hCAP18/LL-37 cathelicidins from the complex media of a mesenchymal-stem-cell factory [294]. The selection of such formulation vehicles and media may also be beneficial to increase the residence time of active ingredients and control their release to protect them from degradation, as shown in the next section.

#### 4.1.5. The Development of Stable and Effective Peptide Formulations

Cathelicidins are prone to degradation by proteolytic enzymes present in biological fluids and tissues. An intuitive alternative to mitigate this challenge would be the use of high-dose formulations that, unfortunately, tend to trigger skin irritation and toxicity. The therapeutically more appropriate methods, as described below, are the combination of antimicrobial agents, the inhibition of protease activity by changing the local pH and by the action of chelating agents, or the chemical modification of either the peptide or the tissue environment [184,185,271,295].

Xia and coauthors have studied the activity against *E. coli* O157:H7 of a combination of cathelicidin BF-15 with thanatin, another antimicrobial peptide, characterized by a β-hairpin structure in its C-terminal region [296]. The authors’ idea was to synergize the membrane disruption ability of both peptides with the inactivation of the New Delhi metallo-β-lactamase by thanatin and the low hemolytic response originating from cathelicidin BF-15. In another illustration, the antimicrobial and biofilm inhibitory activities of the GF-17D3 fragment of LL-37 and a modified scolopendin A2 peptide were tested against *P. aeruginosa*, *S. aureus*, and *Acinetobacter baumannii* [297]. The combination of peptides and antibiotics (i.e., imipenem) showed a high synergetic effect with the lowest minimal inhibitory concentration and reduced the expression of biofilm-related genes for all bacteria.

In terms of increasing the production of cathelicidins in the affected site, the expression of LL-37 can be promoted by fatty acids (in colonic epithelial cells and monocytic cells) [298]. The same can be achieved with gene therapy by transfecting the DNA coding cathelicidins to the cutaneous tissue. For instance, Steinstraeeser et al. have used electroporation to target a plasmid encoding hCAP18/LL-37 to the overall epidermis [299]. In another example, a genetically engineered human skin expressing hCAP18 was developed to provide a sustained release of peptide over burn wounds infected with *A. baumannii* [300].

The modification of cathelicidins’ structure (i.e., α-helix and β-sheet), amino acid composition, or peptide length can impact their stability, antimicrobial activity, and specificity. For this reason, several strategies have been developed, such as modification of net charge or hydrophobicity [301,302,303], or shortening peptide amino acid sequences, suitable for designing synthetic cathelicidins with a selective spectrum of antimicrobial targets with negligible damage to the human microbiome [304].

For instance, a study on LL-37 variants revealed that positively charged residues of LL-37-derived peptides are essential for activity against gram-negative bacteria. In contrast, peptides with higher hydrophobicity are effective against gram-positive bacteria. In addition, one of the studied peptide variants showed reduced toxicity to human cells [305]. A similar study of shortened analogues of porcine cathelicidin PMAP-36, with substituted or lipid-modified amino acid residues, indicated that a stable helical conformation was essential for antibacterial activity, but that an additional increase in helicity and/or hydrophobicity did not change or improve the biological activities of peptides [306]. Nevertheless, introducing a non-proteinogenic α-aminoisobutyric acid instead of leucine improved the proteolytic stability of the peptide.

In addition, the substitution of L-amino acids at four protease cleavage sites by the respective D-residues, improved the proteolytic stability of EFK17, a fragment of LL-37 [307]. Other modifications of EFK17, such as tryptophan substitution at cleavage sites or N-terminal amidation and acetylation, increased not only the proteolytic stability but also the bactericidal activity of the peptide [307]. In the case of the RN15 peptide, a fragment of the cathelicidin MH425517 isolated from *Crocodylus siamensis*, a tryptophan substitution on the non-polar surface of the peptide, was used to enhance its helicity, while a lysine substitution was used to increase the net charge, promoting an inhibitory effect against both gram-positive and gram-negative bacteria [308]. Gunasekera et al. have cyclized dimers of the smallest fragment of LL-37 (i.e., KR-12) with linkers composed of two to four amino acid residues [309]. The dimers showed enhanced proteolytic stability and antimicrobial activity against *P. aeruginosa*, *S. aureus,* and *Candida albicans*, which was followed by an unfortunate increase in hemolytic and cytotoxic activities. Interestingly, stereoisomers of certain naturally occurring peptides, such as D-LL-37, which match the effectiveness of the L-peptide isomer LL-37 in inhibiting biofilm formation, are more resistant to protease degradation [310].

As the relationship between peptide structure and its activity and selectivity is not always straightforward and makes theoretical predictions difficult, the results of these studies provide a basis for designing more selective and effective peptides to treat infections caused by different types of antibiotic-resistant pathogens.

Differently from gene and peptide engineering, another strategy that can be adopted to decrease the degradation of the peptide is delaying its kinetics of release from the formulation. Several colloidal systems, for example, can be used to encapsulate cathelicidins in protective carriers [284,311]. Chereddy et al. showed that poly(lactic-co-glycolic acid) was able to form nanoparticles with LL-37 that presented increased stability and acidified the environment upon release [312]. Additionally, OH-CATH30, a cathelicidin isolated from king cobra, has been encapsulated in carboxymethyl chitosan nanoparticles to obtain improved stability and sustained release [313]. In another example, collagen was explored as a carrier for LL-37, with the study specifically evaluating its stability, antimicrobial activity, and cytotoxicity. The investigation assessed both unmodified LL-37 and its modified variants fused with collagen-binding domains (CBDs) derived from fibronectin (*f*CBD) or collagenase (*c*CBD). Individual peptide variants were incorporated into PURACOL^®^ type I collagen scaffolds, commonly used in wound healing applications. After 2 weeks, up to 99% of LL-37, when modified with the fibronectin-derived CBD, was retained on the scaffold, demonstrating excellent retention properties. Both modified and unmodified LL-37 demonstrated no cytotoxic effects on fibroblasts, even at concentrations far exceeding the cytotoxic threshold observed in solution, and retained either similar antimicrobial activity (*c*CBD-LL-37) or reduced activity (*f*CBD-LL-37) compared to unmodified LL-37. This delivery strategy addresses critical challenges in using antimicrobial peptides for wound healing, including the need for sustained antimicrobial activity and the mitigation of cytotoxicity, ensuring both efficacy and safety at the application site [314].

Proteolysis protection through hindrance or entrapment in polymeric or nanofibrous networks, as shown above, however, can come with a cost. The work of Boge and coauthors epitomizes this struggle [315]. The authors prepared cubosomes from an aqueous liquid crystalline gel of glycerol monooleate and Pluronic F127^®^ that were able to adsorb and protect LL-37 from enzymatic attack, but decreased the antimicrobial effect due to a poor release of the peptide. Interestingly, early work showed that cubosomes of similar composition but different cathelicidin loads may vary in the supramolecular organization—a property known to affect the release profile of actives [316].

Alternatives to obtain an appropriate balance between stability and robust delivery are emulsions and liposomes [311,317,318]. Liposomal formulations, in particular, show advantages because of their characteristic arrangement into a lipidic bilayer phase and an aqueous core. This creates a versatile environment for amphiphilic molecules and improves the biocompatibility upon skin application [319]. Liposome formulations containing distearoyl-phosphatidylcholine and distearoyl-phosphatidylglycerol, for example, were able to entrap the cationic peptide nisin and protect it from extreme pH values and moderate heat upon storage [320]. Still, the method of preparation used by the authors (i.e., rehydration of lipid unilamellar films) involved a complex combination of temperatures ranging from 5 to 60 °C, extrusion through membranes, and size exclusion chromatography.

Indeed, the formulation process itself can lead to the degradation of peptides. The manufacturing of biopharmaceuticals is usually not compatible with high temperatures, high shear, multiple equipment surfaces, and organic solvents. Therefore, there is often a need for stabilizing agents, such as antioxidants, chelators, and antiaggregants [321,322,323]. Also, low-energy methods of production are preferred. One example is the spontaneous emulsification technique and other methods based on the phase inversion phenomenon, which can be used in the preparation of nanoemulsions, solid lipid nanoparticles, and nanostructured lipid carriers. In these techniques, the particle/vesicle association is mostly a result of the physicochemical properties of the mixture of the components and not of the high mechanical and thermal stresses [324,325,326]. Regardless of the technological tools used, the main message is that the successful formulation of cathelicidins targeting skin delivery requires pre-formulation studies of compatibility and the continuous assessment of stability.

### 4.2. Challenges Associated with the Microbial Resistance Mechanisms to Cathelicidins

AMPs, including cathelicidins, were initially believed to be less prone to the development of microbial resistance due to their ability to function through non-specific membrane interaction and multiple mechanisms of action. However, ongoing studies demonstrate that various microorganisms have evolved multiple strategies to diminish the effectiveness of cathelicidins. The common patterns of cathelicidin resistance mechanisms among pathogenic microorganisms typically include altering molecules in the cell envelope to repel these AMPs, proteolytic cleavage of cathelicidins, production of proteins that trap cathelicidins, active pumping of cathelicidins out of the cell using efflux systems, altering cellular processes within the host, and the use of complex gene regulatory networks [327,328].

#### 4.2.1. Resistance Mediated by Cell Surface Modifications

Both gram-positive and gram-negative bacteria can naturally resist cathelicidins by decreasing the negative charge of their cell walls. This natural defense mechanism helps to repel cathelicidins before they reach their targets. Within the group of gram-negative bacteria, alterations to the lipid A segment of LPS seem to diminish the overall negative charge, which in turn helps to counteract the action of cathelicidins. For example, in *P. aeruginosa*, the PmrAB-like signal transduction system is responsible for adding 4-aminoarabinose (Ara4N) to the phosphate group of lipid A, resulting in increased resistance to cathelicidins [329]. Changes in lipid composition associated with increased resistance to cathelicidins also include acylation of lipids, which is believed to decrease the permeability of the outer membrane. For example, in *Haemophilus influenzae*, the presence of phosphorylcholine associated with its LPS portion of the cell surface has been linked to the ability to confer resistance to LL-37 [330]. Increased lipid A acylation as a protection mechanism against LL-37 and mCRAMP has also been observed in *Salmonella enterica* [331]. Yet another resistance mechanism against cathelicidins developed by gram-negative bacteria includes the addition of phosphoethanolamine either to lipid A or to 3-deoxy-D-manno-octulosonic acid, as observed in *Neisseria gonorrhoeae* [332] and *Pasteurella multocida*, respectively [333].

In gram-positive bacteria such as *S. aureus*, an original five-component regulatory system, composed of the glycopeptide resistance-associated proteins GraRSX and the vancomycin resistance-associated VraGF ABC transporter, is involved in the sensing of cathelicidins and signal transduction. The system regulates the surface charge by facilitating the addition of positively charged groups to the bacterial surface. It controls the expression of several key genes, including those involved in the D-alanylation of lipoteichoic acid in the *dlt* operon [334,335,336]. Studies involving mutant *S. aureus* strains with altered genes responsible for D-alanine and L-lysine cell wall modifications have highlighted the importance of this system in resistance to cathelicidins, as these mutants are more susceptible to cathelicidins and are more readily phagocytosed by neutrophils. Nishi et al. reported that mutations in the *LysC* gene, responsible for converting aspartic acid into lysine, alter lysyl-phosphatidylglycerol levels in the *S. aureus* cell membrane, thereby increasing susceptibility to LL-37 [337]. Resistance mechanisms against AMPs based on the action of the *dlt* operon have also been observed in other gram-positive bacteria, such as *Listeria monocytogenes* [338], *Streptococcus agalactiae (*group B *Streptococcus*) [339], and *B. subtilis* [340]. Another possible explanation for increased bacterial resistance to cathelicidins is that D-alanylation decreases the overall negative charge and increases the peptidoglycan layer’s density in the bacterial cell wall, which can impede AMP access to the cell membrane [341]. In *S. agalactiae*, the *PonA* gene encodes penicillin-binding protein 1a (PBP1a), a multifunctional molecule involved in cell wall synthesis. PBP1a plays roles in both the polymerization of glycan chains and the forming of peptide cross-links. *S. agalactiae ponA* mutants exhibit heightened sensitivity to killing by LL-37. Additionally, it has been determined that the PBP1a protein plays a pivotal role in *S. agalactiae* resistance to phagocytic clearance [342]. Further studies indicate that microbial adaptation to cathelicidins is associated with biosynthesis and the cross-linking of the cell envelope. In *S. aureus*, a two-component VraRS system has been identified, playing a crucial role in cathelicidin resistance by enhancing cell wall synthesis, thus thickening it. Disruption of this system increases susceptibility to LL-37, impacting bacterial attachment, biofilm formation, and susceptibility to phagocytosis [336]. Another possible mechanism of bacterial resistance to cathelicidin is the glycosylation of cell wall components. For instance, in *L. monocytogenes*, the glycosylation of teichoic acids in the cell wall protects it against LL-37 and CRAMP [343].

#### 4.2.2. Resistance Mediated by Proteolytic Cleavage

Many gram-positive and gram-negative bacterial human pathogens secrete or display surface proteases that can evade the action of cathelicidins. *Streptococcus pyogenes (*group A *Streptococcus)* releases cysteine proteases, coded by the *SpeB* and *IdeS* genes, which have been shown to cleave LL-37 [344]. Apart from directly degrading cathelicidin, the *SpeB-*encoded protease can indirectly affect these peptides by breaking down tissue proteoglycans. The degradation products of proteoglycans bind to LL-37 through electrostatic interactions, effectively inhibiting it [345]. Other examples of significant human pathogens effectively degrading LL-37 or its synthetic analogue C18G by expressing their proteases include *S. aureus* producing metalloproteinase and V8 protease, encoded by the *Aur* and *SspA* genes, respectively; *S. enterica* producing surface protease (*PgtE* gene); *P. aeruginosa* producing elastase (*LasB* gene); *Proteus mirabilis* producing metalloproteinase (*ZapA* gene); and *Enterococcus faecalis* producing gelatinase (*GelE* gene) [331,344,346]. To surmount the challenge of proteolytic degradation of cathelicidins, and thus enhance their efficacy, innovative strategies involving modification of these AMPs are being explored, as already discussed in Section 4.1.5. In addition to that, some members of the cathelicidin family possess structural properties that render them naturally resistant to the action of bacterial proteases. This is the case for mammalian cathelicidins rich in proline, which are resistant to serine proteases, or the case of Atlantic hagfish cathelicidins, which contain bromotryptophan residues that may reduce the susceptibility of active peptides to proteolysis due to steric factors [347].

#### 4.2.3. Resistance Mediated by Complexation Mechanisms

Bacteria can avoid cathelicidin action through bacterial-anchored or secreted proteins and saccharides, or by induction of the release of molecules from the host cell surface that bind to cathelicidins. *S. aureus* produces an enzyme known as staphylokinase (SK), which facilitates the conversion of plasminogen into its active form, plasmin. Interestingly, SK exhibits a direct affinity for LL-37 and mCRAMP. This interaction appears to enhance the ability of SK to activate plasminogen, which in turn leads to the inactivation of these cathelicidins [348]. Certain strains of the *S. pyogenes* also produce a complement-inhibitory protein called serum inhibitor of complement (SIC), named specifically for its ability to incorporate into the membrane attack complex of complement and inhibit its activity. This protein was initially identified as defending against destruction by the membrane attack complex [349]. Later, it was discovered that SIC is capable of directly binding to LL-37 and inhibiting its antimicrobial effects [350]. Furthermore, *S. pyogenes* contains M surface-anchored protein, which plays multiple roles during infection [351]. M protein is anchored into the bacterial cell wall by its conserved C-terminus. From there, it extends outward as a coiled-coil dimer, creating a fibrous layer over the bacterial surface. Some isolates show resistance to cathelicidins like LL-37, with studies confirming the role of M protein in cathelicidin binding. Serotypes exhibiting elevated AMP resistance are frequently linked to serious diseases [352]. Several pathogenic bacteria produce outer capsules comprising high-molecular-weight polysaccharides, which enhance their in vivo survival and impede the binding of cathelicidins to the bacterial cell surface. For instance, *Campylobacter jejuni* lipooligosaccharide production enhances its resistance to LL-37 [353], mutant *Neisseria meningitidis* serotype B lacking capsules is more susceptible to LL-37 and mCRAMP [354]. The hyaluronan capsule produced by *S. pyogenes* contributes to its survival within neutrophil extracellular traps (NETs) by enhancing its resistance to LL-37 [355]. The primary structural element of NETs consists of nuclear or mitochondrial DNA, which is decorated by granule proteases and cationic antimicrobial proteins such as histones and cathelicidins, with the function of capturing and killing pathogens [356]. Some bacteria, such as *S. pyogenes* [357], *S. agalactiae* [358], *S. pneumoniae* [359], and *S. aureus* [360] can evade NETs by secreting their DNases, which in turn enables them to escape and persist.

#### 4.2.4. Resistance Mediated by Energy-Dependent Export

Energy-driven efflux pumps in bacteria represent yet another resistance mechanism against cathelicidins, based on removing them from the membrane to the extracellular environment. For example, in *N. gonorrhoeae* and *N. meningitidis*, the multiple transferable resistance (Mtr) MtrCDE exporter of the resistance/nodulation/division family contributes to LL-37 and mCRAMP resistance, as supported by various studies [361,362,363,364]. In *Haemophilus ducreyi, the deletion* of *MtrC* increases sensitivity to LL-37 [365]. In *S. pneumoniae*, the macrolide efflux protein E/macrolide efflux-like protein (MefE/Mel) efflux pump represents an inducible resistance mechanism to macrolide antibiotics. It has been shown that LL-37 can alter the expression of the *MefE* and *Mel* genes, leading to increased resistance to LL-37 and mCRAMP, respectively [366]. Regarding ABC transporters, these are often multi-drug exporters with broad substrate specificities that confer resistance to various antimicrobial agents [367]. This is also the case for the transporters of the BceAB group found in staphylococci, which are likely responsible for resistance to antibiotics and AMPs, including the human cathelicidin LL-37 [368].

#### 4.2.5. Resistance Mediated by Alteration of Host AMP Production

Another concept in targeted bacterial survival strategies is based on interference or suppression of host cathelicidin production in response to pathogenic attack. For example, the expression of LL-37 in human intestinal epithelial cells is significantly downregulated at both the transcriptional and translational levels by MxiE, a bacterial transcriptional regulator from *Shigella* that controls a set of virulence effectors located on its plasmid DNA [369]. Another study, published by Chakraborty et al., describes the transcriptional repression of LL-37 in intestinal epithelial cells upon infection caused by *Vibrio cholerae* and enterotoxigenic *E. coli* [370]. Downregulation of LL-37 RNA occurs as a result of the action of the virulence protein cholera toxin and labile toxin, respectively.

#### 4.2.6. Resistance Mediated by Bacterial Regulatory Systems

PhoP/PhoQ (PhoPQ) is one of the most studied bacterial two-component regulatory systems that regulate various genes or gene clusters crucial for bacterial survival and pathogenesis. One of its functions is contributing to the development of antimicrobial resistance. For example, the PhoQ sensor domain of *Salmonella typhimurium* is directly activated by the presence of LL-37 by removing and displacing bound divalent cations, resulting in the transfer of phosphates from PhoQ to PhoP as an initial step in the signal transduction cascade [371,372]. Examples of genes regulated by this sensor kinase include genes for the Ara4N modification of LPS or genes playing a role in acylation or deacylation of lipid A, which make bacteria more resistant to cationic antimicrobial peptides [373]. *P. aeruginosa* possesses a homologous PhoPQ system, which confers resistance to LL-37 and other cathelicidins [374]. PhoPQ homologs in general are present in many gram-negative bacteria indicating their importance in pathogenic adaptation [375,376]. Another example of utilizing global regulatory networks to confer resistance against cathelicidin involves the degradation of NETs by bacterial DNAs, as mentioned above (Section 4.2.3).

Given the adaptability of pathogens, comprehensive knowledge of resistance mechanisms against cathelicidins is essential for developing innovative therapeutic approaches based entirely or partially on the action of antimicrobial peptides (AMPs). These approaches can adapt to the evolutionary changes of pathogens, thereby preserving the efficacy of these peptides and supporting the long-term effectiveness of antimicrobial therapies.

## 5. Overview and Future Perspectives

Cathelicidins hold significant promise as versatile therapeutic agents for various skin conditions, including the treatment of infections, inflammatory management, and the promotion of wound healing and scar reduction (see Table 1). Treatments based on cathelicidins act as “replacement therapies”, supplementing peptides at body sites where endogenous levels are insufficient. This means that the probability of adverse reactions is minimized. Differently from antibiotics, the safety of peptides is enhanced by their degradation into natural amino acids and their short half-life, i.e., preventing unwanted accumulation in tissues [377]. Also, therapeutic peptides are generally less immunogenic than recombinant proteins and antibodies [378] and local administration further reduces the risk of systemic negative effects and toxicity [379].

Considering all these facts, it would be expected that cathelicidins could already be commercially available as a new generation of antibiotics. However, only a few AMPs are currently in clinical use, and none of these are cathelicidins. The reasons for the limited clinical application have been the low metabolic stability due to proteolytic degradation and other issues, such as higher production costs compared to conventional antibiotics [301,380]. Moreover, some safety issues such as potential mutagenicity [381], allergenic properties [382,383], tumorigenic effects [384,385], pro-inflammatory properties [145,386], and hemolytic activity [387] have been reported for cathelicidins and raise points of concern. For instance, the FDA currently categorizes LL-37, one of the most well-known cathelicidins, as a bulk drug substance 503A, category 2. The agency states: “Compounded drugs containing cathelicidin LL-37 may pose a risk for immunogenicity for certain routes of administration and may have complexities concerning peptide-related impurities and active pharmaceutical ingredients characterization. FDA lacks sufficient safety-related information regarding cathelicidin LL-37 to know whether the drug would cause harm when administered to humans. Nonclinical research findings suggest detrimental effects on male reproduction and that this drug can be pro-tumorigenic in some tissues” [388]. In this respect, further research and clinical trials are needed to fully elucidate the clinical potential of not only LL-37 but also other cathelicidins (Table 3).

Additionally, it has been publicly stated by regulatory authorities that developing new antibacterial drugs is problematic because they should be used sparingly to prevent the development of pathogen resistance to a new mechanism of action, which also means that a novel antibiotic may have a very restricted market [389]. This limitation may negatively impact the assessment of the risks of investment slumps, impairing the likelihood of funding expensive research and development costs. Unfortunately, important information about the reasons for the success or failure of therapeutic peptides may become inaccessible if preclinical or clinical trials are discontinued, or if licenses are transferred to other companies.

Beyond being discouraging, our opinion is that this scenario calls for action and that forward movement is possible. Future efforts must combine the basic knowledge accumulated in the areas of molecular biology, biotechnology, and drug delivery to finally translate cathelicidins into medical practice. The key to overcoming current limitations is understanding the mechanisms by which cathelicidins perform their diverse biological functions, particularly how they target and destroy microbial cells. Current bioinformatics tools assist in designing novel cathelicidin-derived peptides with desired features to produce potent drugs with enhanced antimicrobial activity and improved target specificity. Additionally, advanced biotechnological methods enable the mass production of cathelicidins in a stable form in planta at a reasonable cost. Ultimately, a multidisciplinary approach combining the above strategies with tissue-specific and dose-optimized treatment, co-administration with other antimicrobial agents focusing on synergistic combinations, and innovative drug delivery carriers based on nanomaterials, presents a promising pathway toward the development of effective and economically viable cathelicidin-based therapeutics.

## Figures and Tables

**Table 1:** List of natural cathelicidins [1].

Peptide	Source	Activity	Sequence	Reference
	**Mammals**			
LL-37 parent	*Homo sapiens*	Anti-gram+ and gram- antiviral, antifungal, candidacidal, antiparasitic, spermicidal, anti-HIV, chemotactic, anti-MRSA, enzyme inhibitor, anti-TB, anti-sepsis, synergistic AMPs, hemolytic, antibiofilm, wound healing, anticancer	LLGDFFRKSKEKIGKEFKRIVQRIKDFLRNLVPRTES	[18]
ALL-38 (alternatively cleaved form of human LL-37)	*Homo sapiens*	Anti-gram+ and gram-	ALLGDFFRKSKEKIGKEFKRIVQRIKDFLRNLVPRTES	[19]
KS-27 (fragment of LL-37)	*Homo sapiens*	Anti-gram+ and gram-	KSKEKIGKEFKRIVQRIKDFLRNLVPR	[20]
LL-29 (fragment of LL-37)	*Homo sapiens*	Anti-gram+ and gram-	LLGDFFRKSKEKIGKEFKRIVQRIKDFLR	[20]
LL-23 (fragment of LL-37)	*Homo sapiens*	Anti-gram+ and gram-, antifungal	LLGDFFRKSKEKIGKEFKRIVQR	[20]
KR-20 (fragment of LL-37)	*Homo sapiens*	Anti-gram+ and gram-, antifungal, candidacidal, antiparasitic	KRIVQRIKDFLRNLVPRTES	[21]
KS-30 (fragment of LL-37)	*Homo sapiens*	Anti-gram+ and gram-, antifungal, candidacidal	KSKEKIGKEFKRIVQRIKDFLRNLVPRTES	[21]
RK-31 (fragment of LL-37)	*Homo sapiens*	Anti-gram+ and gram-, antifungal, candidacidal, hemolytic	RKSKEKIGKEFKRIVQRIKDFLRNLVPRTES	[21]
TLN-58 (alternatively cleaved form of LL-37)	*Homo sapiens*	Anti-gram+	TLNQARGSFDISCDKDNKRFALLGDFFRKSKEKIGKEFKRIVQRIKDFLRNLVPRTES	[22]
Organgutan ppyLL-37	*Pongo*	Anti-gram+ and gram-	LLGDFFRKAREKIGEEFKRIVQRIKDFLRNLVPRTES	[23]
Gibbon hmdSL-37	*Hylobatidae*	Anti-gram+ and gram-, antifungal, candidacidal	SLGNFFRKARKKIGEEFKRIVQRIKDFLQHLIPRTEA	[23]
pobRL-37	*Cercopithecidae*	Anti-gram+ and gram-, antifungal, candidacidal	RLGNFFRKAKKKIGRGLKKIGQKIKDFLGNLVPRTES	[23]
cjaRL-37	New World monkeys	Anti-gram+ and gram-	RLGDILQKAREKIEGGLKKLVQKIKDFFGKFAPRTES	[23]
RL-37	*Macaca mulatta*	Anti-gram+ and gram-	RLGNFFRKVKEKIGGGLKKVGQKIKDFLGNLVPRTAS	[24]
Bactenecin	*Bos taurus*	Anti-gram+, anti-gram-, synergistic AMPs, wound healing	RLCRIVVIRVCR	[25]
Bactenecin 5	*Bos taurus*	Anti-gram-	RFRPPIRRPPIRPPFYPPFRPPIRPPIFPPIRPPFRPPLGPFP	[26]
Bactenecin 7	*Bos taurus*	Anti-gram-, chemotactic, anti-sepsis	RRIRPRPPRLPRPRPRPLPFPRPGPRPIPRPLPFPRPGPRPIPRPLPFPRPGPRPIPRPL	[26]
BMAP-27	*Bos taurus*	Anti-gram+ and gram-, antifungal, candidacidal, anti-MRSA, hemolytic, antibiofilm, anticancer	GRFKRFRKKFKKLFKKLSPVIPLLHLG	[27]
BMAP-28	*Bos taurus*	Anti-gram+ and gram-, antiviral, antifungal, candidacidal, antiparasitic, anti-MRSA, hemolytic, antibiofilm, anticancer	GGLRSLGRKILRAWKKYGPIIVPIIRIG	[27]
BMAP-34	*Bos taurus*	Anti-gram+ and gram-	GLFRRLRDSIRRGQQKILEKARRIGERIKDIFRG	[28]
Indolicidin	*Bos taurus*	Anti-gram+ and gram-, antiviral, antifungal, anti-HIV, anti-MRSA, hemolytic, antibiofilm, wound healing, anticancer	ILPWKWPWWPWRR	[29]
Bac4	*Bos taurus*	Anti-gram+ and gram-	RRLHPQHQRFPRERPWPKPLSLPLPRPGPRPWPKPL	[30]
BSN-37	*Bos taurus*	Anti-gram+ and gram-	FRPPIRRPPIRPPFYPPFRPPIRPPIFPPIRPPFRPP	[31]
buCATHL4A	*Bubalus bubalis*	Anti-gram+	GLPWILLRWLFFRG	[32]
buCATHL4B	*Bubalus bubalis*	Anti-gram+ and gram-	AIPWIWIWRLLRKG	[32]
buCATHL4C	*Bubalus bubalis*	Anti-gram+ and gram-	RIRFPWPWRWPWWRRVRG	[32]
buCATHL4D	*Bubalus bubalis*	Anti-gram+ and gram-	RIRFPWPWRWPWWPPFRG	[32]
buCATHL4E	*Bubalus bubalis*	Anti-gram+ and gram-	AIPWIWIWWLLRKG	[32]
buCATHL4F	*Bubalus bubalis*	Anti-gram+ and gram-	AIPWSIWWRLLFKG	[32]
buCATHL4G	*Bubalus bubalis*	Anti-gram+ and gram-	AIPWSIWWHLLFKG	[32]
eCATH-1	*Equus asinus*	Anti-gram+ and gram-, antifungal, antiparasitic, anti-MRSA	KRFGRLAKSFLRMRILLPRRKILLAS	[33]
eCATH-2	*Equus asinus*	Anti-gram+ and gram-, antifungal	KRRHWFPLSFQEFLEQLRRFRDQLPFP	[33]
eCATH-3	*Equus asinus*	Anti-gram+ and gram-, antifungal	KRFHSVGSLIQRHQQMIRDKSEATRHGIRIITRPKLLLAS	[33]
EA-CATH1	*Equus asinus*	Anti-gram+ and gram-, antifungal	KRRGSVTTRYQFLMIHLLRPKKLFA	[34]
Tritrpticin	*Sus scrofa*	Anti-gram+ and gram-, antifungal, hemolytic, anticancer	VRRFPWWWPFLRR	[35]
Protegrin 1	*Sus scrofa*	Anti-gram+, antiviral, antifungal, candidacidal, anti-HIV, anti-MRSA, anti-sepsis, synergistic AMPs, antibiofilm	RGGRLCYCRRRFCVCVGR	[36]
Protegrin 2	*Sus scrofa*	Anti-gram+ and gram-, antiviral, antifungal, candidacidal	RGGRLCYCRRRFCICV	[37]
Protegrin 3	*Sus scrofa*	Anti-gram+ and gram-, antiviral, antifungal, candidacidal	RGGGLCYCRRRFCVCVGR	[38]
Protegrin 4	*Sus scrofa*	Anti-gram+ and gram-, antiviral	RGGRLCYCRGWICFCVGR	[39]
Protegrin 5	*Sus scrofa*	Anti-gram+ and gram-, antiviral, antifungal, candidacidal	RGGRLCYCRPRFCVCVGR	[40]
PMAP-23	*Sus scrofa*	Anti-gram+ and gram-, antifungal, candidacidal	RIIDLLWRVRRPQKPKFVTVWVR	[41]
PMAP-36	*Sus scrofa*	Anti-gram+ and gram-	VGRFRRLRKKTRKRLKKIGKVLKWIPPIVGSIPLGCG	[41]
PMAP-37	*Sus scrofa*	Anti-gram+ and gram-	GLLSRLRDFLSDRGRRLGEKIERIGQKIKDLSEFFQS	[42]
PR-39	*Sus scrofa*	Anti-gram+ and gram-, wound healing, anticancer	RRRPRPPYLPRPRPPPFFPPRLPPRIPPGFPPRFPPRFP	[43]
Prophenin-1	*Sus scrofa*	Anti-gram+ and gram-	AFPPPNVPGPRFPPPNFPGPRFPPPNFPGPRFPPPNFPGPRFPPPNFPGPPFPPPIFPGPWFPPPPPFRPPPFGPPRFP	[44]
Prophenin-2	*Sus scrofa*	Anti-gram+ and gram-	AFPPPNVPGPRFPPPNVPGPRFPPPNFPGPRFPPPNFPGPRFPPPNFPGPPFPPPIFPGPWFPPPPPFRPPPFGPPRFP	[45]
PR-35	*Sus scrofa*	Anti-gram-	RPPYLPRPRPPPFFPPRLPPRIPPGFPPRFPPRFP	[46]
Cyclic dodecapeptide	*Ovis aries*	Anti-gram+ and gram-	RICRIIFLRVCR	[47]
SMAP-29	*Ovis aries*	Anti-gram+ and gram-, antifungal, candidacidal, anti-MRSA, anti-sepsis, hemolytic, antibiofilm	RGLRRLGRKIAHGVKKYGPTVLRIIRIAG	[47,48]
SMAP-34	*Ovis aries*	Anti-gram+ and gram-, anti-MRSA	GLFGRLRDSLQRGGQKILEKAERIWCKIKDIFR	[49]
OaBac5	*Ovis aries*	Anti-gram+ and gram-, anti-sepsis	RFRPPIRRPPIRPPFRPPFRPPVRPPIRPPFRPPFRPPIGPFP	[30]
OaBac6	*Ovis aries*	Anti-gram+ and gram-	RRLRPRHQHFPSERPWPKPLPLPLPRPGPRPWPKPLPLPLPRPGLRPWPKPL	[50]
OaBac7.5	*Ovis aries*	Anti-gram+ and gram-, anti-sepsis	RRLRPRRPRLPRPRPRPRPRPRSLPLPRPQPRRIPRPILLPWRPPRPIPRPQIQPIPRWL	[30]
OaBac11	*Ovis aries*	Anti-gram+ and gram-	RRLRPRRPRLPRPRPRPRPRPRSLPLPRPKPRPIPRPLPLPRPRPKPIPRPLPLPRPRPRRIPRPLPLPRPRPRPIPRPLPLPQPQPSPIPRPL	[30]
OaBac5gamma	*Ovis aries*	Anti-gram+ and gram-, antifungal, anti-MRSA	RFRPPILRPPIRPPFRPPFRPPVRPPIRPPFRPPFRPPIGPFP	[30]
mini-ChBac7.5Nalpha	*Capra hircus*	Anti-gram+ and gram-, antifungal, candidacidal, anti-sepsis	RRLRPRRPRLPRPRPRPRPRPR	[51]
mini-ChBac7.5Nbeta	*Capra hircus*	Anti-gram+ and gram-, anti-sepsis	RRLRPRRPRLPRPRPRPRPRP	[51]
ChBac3.4	*Capra hircus*	Anti-gram+ and gram-, antifungal, hemolytic, anticancer	RFRLPFRRPPIRIHPPPFYPPFRRFL	[52]
ChBac5	*Capra hircus*	Anti-gram+ and gram-, anti-sepsis	RFRPPIRRPPIRPPFNPPFRPPVRPPFRPPFRPPFRPPIGPFP	[53]
VicBac	*Vicugna pacos*	Anti-gram+ and gram-, anti-inflammatory	RRIRRPRLPRPRVPRPRIPPRIPRPVLPPPRVPFPRFPR	[54]
P9	*Cervus elaphus*	Anti-gram+ and gram-, antifungal	RFIPPILRPPVRPPFRPPFRPPFRPPPIIRFFGG (incomplete)	[55]
Cathelicidin-AM	*Ailuropoda melanoleuca*	Anti-gram+ and gram-, antifungal	GRLRNLIEKAGQNIRGKIQGIGRRIKDILKNLQPRPQV	[56]
K9CATH	*Canis lupus*	Anti-gram+ and gram-, antifungal, candidacidal, anti-sepsis	RLKELITTGGQKIGEKIRRIGQRIKDFFKNLQPREEKS	[57]
Saha-CATH3	*Sarcophilus harrisii*	Antifungal, anticancer	KRMGIFHLFWAGLRKLGNLIKNKIQQGIENFLG	[58]
Saha-CATH5	*Sarcophilus harrisii*	Anti-gram+ and gram-, antifungal, anti-MRSA, anticancer	KRIGLIRLIGKILRGLRRLG	[58]
Saha-CATH6	*Sarcophilus harrisii*	Anti-gram+ and gram-, antifungal, anticancer	KRIRFFERIRDRLRDLGNRIKNRIRDFFS	[58]
FeCath	*Felis catus*	Anti-gram+ and gram-	QLGELIQQGGQKIVEKIQKIGQRIRDFFSNLRPRQEA	[59]
WAM1	*Macropus eugenii*	Anti-gram+ and gram-	KRGFGKKLRKRLKKFRNSIKKRLKNFNVVIPIPLPG	[60]
WAM2	*Macropus eugenii*	Anti-gram+ and gram-	KRGLWESLKRKATKLGDDIRNTLRNFKIKFPVPRQG	[60]
MaeuCath7	*Macropus eugenii*	Anti-gram-	KRGLWESLKRKVTKLGDDIRNTLRNFKIKFPVPRQG	[61]
Taac-CATH1	*Tachyglossus aculeatus*	Anti-gram+ and gram-, anti-MRSA	PIRTKRRWKLIKKGGKIVKDLLTKNNIIILPGGNE	[61]
ModoCath1	*Monodelphis domestica*	Anti-gram+ and gram-	VKRTKRGARRGLTKVLKKIFGSIVKKAVSKGV	[62]
ModoCath4	*Monodelphis domestica*	Anti-gram+ and gram-, anti-MRSA	SKTKRRSLLKRLGDGIRGFWNGFRGRK	[61]
ModoCath5	*Monodelphis domestica*	Anti-gram+ and gram-, antiviral	WYQLIRTFGNLIHQKYRKLLEAYRKLRD	[62]
ModoCath6	*Monodelphis domestica*	Anti-gram-	VRRSKRGIKVPSFVKKVLKDVVSESIS	[62]
PhciCath5	*Phascolarctos cinereus*	Anti-gram+ and gram-, antifungal, anti-MRSA	KRGGIWKLIRPLGRGAGRILRHFHIDFCGNC	[63]
PAM1	*Ornithorhynchus anatinus*	Anti-gram+ and gram-	RTKRRIKLIKNGVKKVKDILKNNNIIILPGSNEK	[60]
PAM2	*Ornithorhynchus anatinus*	Anti-gram+ and gram-	RPWAGNGSVHRYTVLSPRLKTQ	[60]
HA-CATH	*Hipposideros armiger*	Anti-gram+ and gram-, antifungal	ILGRLRDLLRRGGRKIGQGLERIGQRIQGFFSNREPMEES	[64]
MI-LN-35	*Myotis lucifugus*	Anti-gram+ and gram-, antifungal, candidacidal	LNPLIKAGIFILKHRRPIGRGIEITGRGIKKFFSK	[64]
PD-CATH	*Phyllostomus discolor*	Anti-gram+ and gram-, antifungal, candidacidal	ILGPALRIGGRIAGRIAGKLIGDAINRHRERNRQRRG	[64]
To-KL37	*Talpa occidentalis*	Anti-gram+ and gram-	KLFGKVGNLLQKGWQKIKNIGRRIKDFFRNIRPMQEA	[65]
Hg-CATH	*Heterocephalus glaber*	Anti-gram-	RRFRRTVGLSKFFRKARKKLGKGLQKIKNVLRKYLPRPQYAYA	[66]
mCRAMP	*Mus musculus*	Anti-gram+ and gram-, antifungal, candidacidal, antibiofilm, anticancer	GLLRKGGEKIGEKLKKIGQKIKNFFQKLVPQPEQ	[67]
rCRAMP	*Rattus rattus*	Anti-gram+ and gram-, anti-MRSA	GLVRKGGEKFGEKLRKIGQKIKEFFQKLALEIEQ	[49]
CAP18 (106–142)	*Oryctolagus cuniculus*	Anti-gram+ and gram-, anti-MRSA, anti-sepsis	GLRKRLRKFRNKIKEKLKKIGQKIQGFVPKLAPRTDY	[68]
TC-33	*Tupaia belangeri chinensis*	Anti-gram+ and gram-	LLRRGGEKLAEKFEKIGQKIKNFFRKLLPETES	[69]
CAP11	*Cavia porcellus*	Anti-gram+ and gram-, antiviral, anti-sepsis	GLRKKFRKTRKRIQKLGRKIGKTGRKVWKAWREYGQIPYPCRI	[70]
BM-CATH	*Balaenoptera musculus*	Anti-gram+ and gram-	GRFSRLRKRIRKVWRKIGPIAGPIIGHFG	[71]
LV-CATH	*Lipotes vexillifer*	Anti-gram+ and gram-	GRFRRLRNRIRNIWRKIGPIAGPLISRFG	[71]
	**Birds**			
Chicken CATH-1	*Gallus galllus*	Anti-gram+ and gram-, anti-MRSA, anti-inflammatory, anti-sepsis	RVKRVWPLVIRTVIAGYNLYRAIKKK	[72]
Chicken CATH-2	*Gallus galllus*	Anti-gram+ and gram-, anti-MRSA, anti-sepsis, hemolytic, antibiofilm	RFGRFLRKIRRFRPKVTITIQGSARFG	[73]
Chicken CATH-3	*Gallus galllus*	Anti-gram+ and gram-, anti-sepsis	RVKRFWPLVPVAINTVAAGINLYKAIRRK	[72]
Cath-B1	*Gallus gallus*	Anti-gram+ and gram-, antiviral	PITYLDAILAAVRLLNQRISGPCILRLREAQPRPGWVGTLQRRREVSFLVEDGPCPPGVDCRSCEPGALQHCVGTVSIEQQPTAELRCRPLRPQ	[74]
Cl-CATH2	*Columba livia*	Anti-gram+ and gram-, antifungal, candidacidal, anti-inflammatory	LIQRGRFGRFLGRIRRFRPRINFDIRARGSIRLG	[75]
dCATH	*Anas platyrhynchos*	Anti-gram+ and gram-, anti-inflammatory, anti-sepsis, hemolytic	KRFWQLVPLAIKIYRAWKRR	[76]
Pc-CATH1	*Phasianus colchicus*	Anti-gram+ and gram-, antifungal, candidacidal	RIKRFWPVVIRTVVAGYNLYRAIKKK	[77]
cc-CATH2	*Coturnix coturnix*	Anti-gram+ and gram-, antifungal	LVQRGRFGRFLKKVRRFIPKVIIAAQIGSRFG	[78]
cc-CATH3	*Coturnix coturnix*	Anti-gram+ and gram-, antifungal	RVRRFWPLVPVAINTVAAGINLYKAIRRK	[78]
CATH-2	*Corvus splendens*	Anti-gram+ and gram-	LIQRGRFGRFLGKIRHFRPRVKFNVHLRGSVGLG	[79]
	**Fish**			
CodCath	*Gadus morhua*	Anti-gram+ and gram-, antifungal	SRSGRGSGKGGRGGSRGSSGSRGSKGPSGSRGSSGSRGSKGSRGGRSGRGSTIAGNGNRNNGGTRTA	[80]
aCATH	*Plecoglossus altivelis*	Anti-gram-	RMRRSKSGKGSGGSKGSGSKGSKGSKGSGSKGSGSKGGSRPGGGSSIAGGGSKGKGGTQTA	[81]
CATH_BRALE	*Brachymystax lenok*	Anti-gram+ and gram-, antifungal, candidacidal	RRSKARGGSRGSKMGRKDSKGGSRGRPGSGSRPGGGSSIAGASRGDRGGTRNA	[82]
HFIAP-1	*Myxine glutinosa*	Anti-gram+ and gram-	GFFKKAWRKVKHAGRRVLDTAKGVGRHYVNNWLNRYR	[83]
HFIAP-3	*Myxine glutinosa*	Anti-gram+ and gram-	GWFKKAWRKVKNAGRRVLKGVGIHYGVGLI	[83]
rtCATH_1	*Oncorhynchus mykiss*	Anti-gram+ and gram-	RICSRDKNCVSRPGVGSIIGRPGGGSLIGRPGGGSVIGRPGGGSPPGGGSFNDEFIRDHSDGNRFA	[84]
rtCATH-1a	*Oncorhynchus mykiss*	Anti-gram+ and gram-	RRSKVRICSRGKNCVSRLGGGSIIGRPGGGSLIGRPGGGSVIGRPGGGSPPGGGSFNDEFIRDHSDGNRFA	[85]
rtCATH-1b	*Oncorhynchus mykiss*	Anti-gram+ and gram-	RRSKVRICSRGKNCVSRPGGGSVIGRPGGGSPPGGGSFNDEFIRDHSDGNRFA	[85]
rtCATH-1c	*Oncorhynchus mykiss*	Anti-gram+ and gram-	RRSKVRICSRGKNCVSRPGGGSFNDEFIRDHSDGNRFA	[85]
rtCATH-1d	*Oncorhynchus mykiss*	Anti-gram+ and gram-	RRSKVRICSRGKNCVSFNDEFIRDHSDGNRFA	[85]
rtCATH-2a	*Oncorhynchus mykiss*	Anti-gram+ and gram-	RRGKDSGGPKMGRKDSKGCWRGRPGSGSRPGFGSGIAGASGVNHVGTLPASNSTTHPLDNCKISPQ	[85]
rtCATH-2b	*Oncorhynchus mykiss*	Anti-gram+ and gram-	RRGKDSGGPKMGRKDSKGCWRGRPGSGSRPGFGSGIAGASGVNHVGTLPA	[85]
	**Reptiles**			
OH-CATH	*Ophiophagus hannah*	Anti-gram+ and gram-, enzyme inhibitor	KRFKKFFKKLKNSVKKRAKKFFKKPRVIGVSIPF	[86]
BF-CATH	*Bungarus fasciatus*	Anti-gram+ and gram-	KRFKKFFKKLKKSVKKRAKKFFKKPRVIGVSIPF	[86]
NA-CATH	*Naja atra*	Anti-gram+ and gram-, antibiofilm	KRFKKFFKKLKNSVKKRAKKFFKKPKVIGVTFPF	[86]
Hc-CATH	*Hydrophis cyanocinctus*	Anti-gram+ and gram-, antiviral, antifungal, candidacidal, anti-inflammatory, anti-sepsis, antibiofilm	KFFKRLLKSVRRAVKKFRKKPRLIGLSTLL	[87]
Batroxicidin	*Bothrops atrox*	Anti-gram+ and gram-, antiparasitic	KRFKKFFKKLKNSVKKRVKKFFRKPRVIGVTFPF	[88]
Crotalicidin	*Crotalus durissus terrificus*	Anti-gram+ and gram-, antifungal, anticancer, hemolytic	KRFKKFFKKVKKSVKKRLKKIFKKPMVIGVTIPF	[88]
Cathelicidin-BF	*Bungarus fasciatus*	Anti-gram+ and gram-, antifungal, candidacidal, enzyme inhibitor, anticancer	KFFRKLKKSVKKRAKEFFKKPRVIGVSIPF	[89]
CATHPb1	*Python bivittatu*	Anti-gram+ and gram-, antifungal, candidacidal, anti-MRSA, anti-inflammatory, antibiofilm	KRFKKFFRKIKKGFRKIFKKTKIFIGGTIPI	[90]
CATHPb2	*Python bivittatu*	Anti-gram+ and gram-, antifungal, anti-MRSA	KRNGFRKFMRRLKKFFAGGGSSIAHIKLH	[90]
CATHPb4	*Python bivittatu*	Anti-gram+ and gram-, antifungal, anti-MRSA	TRSRWRRFIRGAGRFARRYGWRIALGLVG	[90]
TS-CATH	*Thamnophis sirtalis*	Anti-gram+ and gram-, anti-inflammatory, wound healing	KRFKKFFKKIKKSVKKRVKKLFKKPRVIPISIPF	[71]
SA-CATH	*Sinonatrix annularis*	Anti-gram+ and gram-, antifungal, candidacidal, anti-inflammatory, antibiofilm	KFFKKLKKSVKKHVKKFFKKPKVIGVSIPF	[91]
Aquiluscidin	*Crotalus aquilus*	Anti-gram+ and gram-	KRFKKFFKKVKKSVKKRLKKIFKKPMVIGVSFPF	[92]
RG-29	*Gekko japonicus*	Anti-gram+ and gram-	RWRRFWGKAKRGIKKHGVSIALAALRLRG	[93]
Gj-CATH2	*Gekko japonicus*	Anti-gram+ and gram-, anti-MRSA	RRGIKKFIKKVKKVKKAIKEGIKKGIKKLLSGGGSNIAHGPGGRRHIA	[94]
Ps-CATH4	*Pelodiscus sinensis*	Anti-gram+ and gram-	TRGRWGRFKRRAGRFIRRNRWQIISTGLKLIG	[95]
Ps-CATH6	*Pelodiscus sinensis*	Anti-gram+ and gram-, candidacidal	KKPSKKPKPQAMTFPKVTVEYFPASFSTAALTVPED	[95]
Cm-CATH1	*Chelonia mydas*	Anti-gram+ and gram-, antifungal, candidacidal	RRSIFRKLRRKIKKGLKKGIQHLLAGGRQGLPQGGRPGMI	[96]
Cm-CATH2	*Chelonia mydas*	Anti-gram+ and gram-, antifungal, candidacidal, anti-inflammatory, antibiofilm	RRSRFGRFFKKVRKQLGRVLRHSRITVGGRMRF	[96]
Cm-CATH3	*Chelonia mydas*	Anti-gram+ and gram-, antifungal, candidacidal	TRGRWKRFWRGAGRFFRRHKEKIIRAAVDIVLS	[96]
Cm-CATH4	*Chelonia mydas*	Anti-gram+ and gram-, antifungal, candidacidal	MAFPFSTQRINPEIEEGNASLADLPVTHAGSLPGIKAQVRTALGIALLLVA	[96]
As-CATH4	*Alligator sinensis*	Anti-gram+ and gram-, antifungal, candidacidal, anti-sepsis	RRGLFKKLRRKIKKGFKKIFKRLPPVGVGVSIPLAGRR	[97]
As-CATH5	*Alligator sinensis*	Anti-gram+ and gram-, antifungal, candidacidal, anti-sepsis	TRRKFWKKVLNGALKIAPFLLG	[97]
As-CATH6	*Alligator sinensis*	Anti-gram+ and gram-, anti-sepsis	TRWLWLLRGGLKAAGWGIRAHLNRNQ	[97]
As-CATH7	*Alligator sinensis*	Anti-gram+ and gram-	KRVNWRKVGRNTALGASYVLSFLG	[98]
As-CATH8	*Alligator sinensis*	Anti-gram+ and gram-	KRVNWAKVGRTALKLLPYIFG	[98]
AM-CATH36	*Alligator mississippiensis*	Anti-gram+ and gram-	GLFKKLRRKIKKGFKKIFKRLPPIGVGVSIPLAGKR	[99]
Gg-CATH5	*Gavialis gangeticus*	Anti-gram+ and gram-	TRRKWWKKVLNGAIKIAPYILD	[98]
Gg-CATH7	*Gavialis gangeticus*	Anti-gram+ and gram-	KRVNWRKVGLGASYVMSWLG	[98]
	**Amphibians**			
Cathelicidin-AL	*Amolops loloensis*	Anti-gram+ and gram-, antifungal, candidacidal, anti-MRSA	RRSRRGRGGGRRGGSGGRGGRGGGGRSGAGSSIAGVGSRGGGGGRHYA	[100]
Cathelicidin-PY	*Paa yunnanensis*	Anti-gram+ and gram-, antifungal, candidacidal, anti-inflammatory, anti-sepsis	RKCNFLCKLKEKLRTVITSHIDKVLRPQG	[101]
Lf-CATH1	*Limnonectes fragilis*	Anti-gram+ and gram-, antifungal, candidacidal	PPCRGIFCRRVGSSSAIARPGKTLSTFITV	[102]
Lf-CATH2	*Limnonectes fragilis*	Anti-gram+ and gram-, antifungal, candidacidal	GKCNVLCQLKQKLRSIGSGSHIGSVVLPRG	[102]
Cathelicidin-RC1	*Rana catesbeiana*	Anti-gram+ and gram-, antifungal, candidacidal	KKCKFFCKVKKKIKSIGFQIPIVSIPFK	[103]
Cathelicidin-RC2	*Rana catesbeiana*	Anti-gram+	KKCGFFCKLKNKLKSTGSRSNIAAGTHGGTFRV	[103]
BG-CATH37	*Bufo bufo gargarizans*	Anti-gram+ and gram-	SSRRPCRGRSCGPRLRGGYTLIGRPVKNQNRPKYMWV	[104]
Cathelicidin-PP	*Polypedates puerensis*	Anti-gram+ and gram-, antifungal, anti-inflammatory, anti-sepsis	ASENGKCNLLCLVKKKLRAVGNVIKTVVGKIA	[105]
OL-CATH2	*Odorrana livida*	Anti-gram+ and gram-, anti-inflammatory, anti-sepsis	RKCNFLCKVKNKLKSVGSKSLIGSATHHGIYRV	[106]
HR-CATH	*Hoplobatrachus rugulosus*	Anti-gram+ and gram-, chemotactic	ASKKGKCNLLCKLKQKLRSVGAGTHIGSVVLKG	[107]
Cath-MH	*Microhyla heymonsi Vogt*	Anti-gram+ and gram-, antifungal, candidacidal, anti-inflammatory, enzyme inhibitor	APCKLGCKIKKVKQKIKQKLKAKVNAVKTVIGKISEHLG	[108]
PN-CATH1	*Pelophylax nigromaculata*	Anti-gram+ and gram-, antioxidant	KKCNFFCKLKKKVKSVGSRNLIGSATHHHRIYRV	[109]
PN-CATH2	*Pelophylax nigromaculata*	Anti-gram+ and gram-, antioxidant, anti-inflammatory, anti-sepsis	EGCNILCLLKRKVKAVKNVVKNVVKSVVG	[109]
Cathelicidin-PR1	*Paa robertingeri*	Anti-gram+ and gram-, antifungal, candidacidal	RKCNLFCKAKQKLKSLSSVIGTVVHPPRG	[110]
Ll-CATH	*Leptobrachium liui*	Anti-gram+ and gram-, chemotactic, anti-inflammatory	SRPCNCRCCYVARGNGRCLLRPGCFTVAARPNRSV	[111]
QS-CATH	*Quasipaa spinosa*	Anti-gram+ and gram-, chemotactic	ANRKPPCRGIFCRRVGSGSLIGRPAKDSSNNLSPFIAV	[112]
Cathelicidin-NV	*Nanorana ventripunctata*	Wound healing	ARGKKECKDDRCRLLMKRGSFSYV	[113]
Nv-CATH	*Nanorana ventripunctata*	Anti-gram+ and gram-, anti-inflammatory, anti-sepsis	NCNFLCKVKQRLRSVSSTSHIGMAIPRPRG	[114]
Zs-CATH	*Zhangixalus smaragdinus*	Anti-gram+ and gram-, anti-inflammatory	ASKKGKCNFMCKVKQKLRAIGSKTVIGTVVHKI	[115]
Cathelicidin-DM	*Duttaphrynus melanostictus,* or *Bufo bufo gargarizans Cantor*	Anti-gram+ and gram-, wound healing	SSRRKPCKGWLCKLKLRGGYTLIGSATNLNRPTYVRA	[116]
Cathelicidin-Bg	*Bufo gargarizans*	Anti-gram+	RPCRGRSCSPWLRGAYTLIGRPAKNQNRPKYMWV	[117]
AdCath	*Andrias davidianus*	Anti-gram+ and gram-, anti-sepsis	RPKKVQGRKAEKDNGDGTTAANASGKKKSSNVFK	[118]

**Table 2:** Skin variations across animal models, gender, and body location.

Animal Model		Thickness Epidermis+Dermis	Hair Follicle		
		Female/Male (mm)	Density (/cm^2^)	Diameter (µm)	Hair Section (µm)
Human	Thigh	1.50/1.72 ^I^	14–32 ^IV^	66–170 ^IV^	16–29 ^IV^
	Waist	1.92/1.99 ^I^			
	Deltoid	1.96/2.26 ^I^			
Porcine	Ear	1.95 ^II^	20 ^II^	200 ^II^	82 ^II^
Mouse	Dorsal	0.20–0.25/0.35–0.40 ^III^	5045 ^V^	24 ^V^	-

^I^ Laurent et al.; measured via ultrasound echography [171]. Data were averaged among individuals of 18–70 years old. The values are in accordance with other studies, like in Granieri et al. [172]. ^II^ Jacobi et al.; measured via histology analyses with optical microscopy [177]. No information on gender was provided. ^III^ Calabro et al.; measured via histology analyses with optical microscopy (CD1 mice) [178]. ^IV^ Otberg et al.; measured via histology analyses with optical microscopy [179]. Data were averaged between female and male individuals and the range of values characterizes different areas of the body. ^V^ Mangelsdorf et al.; measured via histology analyses with optical microscopy [180]. Data were collected from female individuals, in the scapular area.

**Table 3:** Clinical trials of cathelicidin-based drugs [190].

Peptide	Target	Status in 2024	Phase	Article/Reference	Clinical Trial ID
LL-37	Melanoma	Completed	I/II	M.D. Anderson Cancer Center, Houston, TX, USA. Induction of antitumor response in melanoma patients using the antimicrobial peptide LL-37	NCT02225366
LL-37	Diabetic foot ulcer	Unknown	II	[191]; Fakultas Kedokteran Universitas Indonesia, Kenari, Indonesia	NCT04098562
OP-145 (AMP60.4Ac) synthetic, LL-37 derived peptide	Otitis media	Completed	II	[192]; OctoPlus BV, Heerenveen, Netherlands	ISRCTN12149720
Recombinant LL-37	COVID-19	Completed, another undefined phase ongoing until 2026	II	[193]; Chinese PLA General Hospital, Beijing, China	ChiCTR2300067840
Synthetic LL-37 (Ropocamptide)	Hard-to-heal venous leg ulcers	Completed	II	[194,195]; Lipopeptide AB (Promore Pharma in present, Solna, Sweden)	EUCTR2012-002100-41-SE, EUCTR2018-000536-10-PL
Omiganan (MBI 226, synthetic analogue of bovine indolicidin)	Evaluate the safety of topical application of AMPs	Completed	I	Mashhad University of Medical Sciences, Mashhad, Iran. Evaluate the safety, side effects, and maximum tolerable dose of 5 AMPs on the skin of healthy volunteers to the treatment of skin and soft tissue infections	IRCT20190924044863N1
Omiganan (MBI 226, synthetic analogue of bovine indolicidin)	Facial seborrheic dermatitis	Ongoing	II	[196]; Maruho Co., Ltd., Osaka, Japan.	NCT03688971
Omiganan (MBI 226, synthetic analogue of bovine indolicidin)	Atopic dermatitis	Completed	II	[197,198]; Cutanea Life Sciences, Wayne, PA, USA	NL-OMON42963
Omiganan (MBI 226, synthetic analogue of bovine indolicidin)	Venous catheterization	Completed	III	[199]	NCT00027248 (BioWest Therapeutics Inc., Vancouver, BC, Canada), NCT00231153 (Mallinckrodt, St. Louis, MO, USA)
Omiganan (MBI 226, synthetic analogue of bovine indolicidin)	Healthy volunteers	Ongoing	II	[200]; Cutanea Life Sciences, Wayne, PA, USA	EUCTR2016-004702-34-NL
Omiganan (MBI 226, synthetic analogue of bovine indolicidin)	Genital warts	Completed	II	[201]; Cutanea Life Sciences, Wayne, PA, USA	NCT02849262
Omiganan (MBI 226, synthetic analogue of bovine indolicidin)	Papulopustular rosacea	Completed	III	Maruho Co., Ltd., Osaka, Japan. Study to evaluate the safety and efficacy of a once-daily CLS001 topical gel versus vehicle.	NCT02576860
Omiganan (MBI 226, synthetic analogue of bovine indolicidin)	Severe acne vulgaris	Completed	II	Cutanea Life Sciences, Inc., Wayne, PA, USA. A study to evaluate the safety and efficacy of Omiganan (CLS001) topical gel versus vehicle in female subjects with moderate-to-severe acne vulgaris.	NCT02571998
Omiganan (MBI 226, synthetic analogue of bovine indolicidin)	Severe acne vulgaris	Completed	II	(a) BioWest Therapeutics Inc., Vancouver, BC, Canada. Safety and efficacy of MBI 226 2.5% and 5.0% topical acne solutions in the treatment of acne; (b) safety and efficacy of MBI 226 1.25% and 2.5% topical acne solutions in the treatment of acne.	a) NCT00211523; b) NCT00211497
Omiganan (MBI 226, synthetic analogue of bovine indolicidin)	Vulvar intraepithelial neoplasia	Completed	II	[201]; Cutanea Life Sciences, Wayne, PA, USA.	EUCTR2015-002724-16-NL
Omiganan (MBI 226, synthetic analogue of bovine indolicidin)	Healthy adult subject	Completed	III	Mallinckrodt, St. Louis, MO, USA. Study of antimicrobial activity of omiganan 1% gel vs. chlorhexidine 2% for topical skin anti-sepsis in healthy adult subjects.	NCT00608959
SGX942 (Dusquetide, synthetic peptide derived from indolicidin)	Treatment of oral mucositis	Ongoing	III	[202,203]	EUCTR2017-003702-41-FR
SGX945 (Dusquetide, synthetic peptide derived from indolicidin)	Aphthous ulcers in Behçet’s disease	Ongoing	II	[203]	NCT06386744
Murepavadin (POL7080; synthetic derivative of pigs protegrin-1)	Pneumonia due to *P. aeruginosa*	Terminated	III	Polyphor Ltd. (Spexis at present, Allschwil, Switzerland)	CTRI/2019/04/018855, NCT03582007, NCT03409679
Murepavadin (POL7080; synthetic derivative of pigs protegrin-1)	Nosocomial pneumonia due to *P. aeruginosa*	Ongoing	III	[204,205] preceding trial NCT02110459; Polyphor Ltd. (Spexis at present, Allschwil, Switzerland)	EUCTR2018-001159-11-FR, EUCTR2018-001159-11-CZ, EUCTR2017-003933-27-HU
Iseganan (IB-367, synthetic analog of protegrin-1)	Pneumonia	Terminated	II/III	[206]; IntraBiotics Pharmaceuticals Inc., Mountain View, CA, USA (closed)	NCT00118781
Iseganan (IB-367, synthetic analog of protegrin-1)	Oral mucositis	Unknown	III	[207]; National Cancer Institute, Bethesda, MD, USA (NCI)	NCT00022373
